# Pathomics Signature for Prognosis and CA19‐9 Interception in Pancreatic Ductal Adenocarcinoma: A Real‐Life, Multi‐Center Study

**DOI:** 10.1002/advs.202515952

**Published:** 2026-01-20

**Authors:** Qiangda Chen, Zhihang Xu, Yiping Zou, Zhenlai Jiang, Yecheng Li, Taochen He, Hanlin Yin, Jiali Li, Yanfei An, Jiande Han, Yuqi Xie, Wei Gan, Yaolin Xu, Wenquan Wang, Junyi He, Haibo Wang, Wenchuan Wu, Zhenyu Ye, Wenhui Lou, Jihui Hao, Liang Liu, Jun Yu, Ning Pu

**Affiliations:** ^1^ Department of Pancreatic Surgery Zhongshan Hospital Fudan University Shanghai China; ^2^ Department of Hepatobiliary Pancreatic Surgery The First Affiliated Hospital of Fujian Medical University Fuzhou China; ^3^ Cancer Center Zhongshan Hospital Fudan University Shanghai China; ^4^ Pancreas Center Tianjin Medical University Cancer Institute and Hospital National Clinical Research Center for Cancer State Key Laboratory of Druggability Evaluation and Systematic Translational Medicine Tianjin Key Laboratory of Digestive Cancer Tianjin's Clinical Research Center for Cancer Tianjin China; ^5^ Department of General Surgery The Second Affiliated Hospital of Soochow University Suzhou China; ^6^ Department of Pathology Zhongshan Hospital, Fudan University Shanghai China; ^7^ Medical College of Yangzhou University Yangzhou China; ^8^ Key Laboratory of Gastrointestinal Cancer (Fujian Medical University), Ministry of Education School of Basic Medical Sciences Fujian Medical University Fuzhou China

**Keywords:** deep learning, pancreatic cancer, pathological character, pathomics, prognosis

## Abstract

Histopathological hematoxylin and eosin (H&E) slides contain valuable prognostic information for pancreatic ductal adenocarcinoma (PDAC), yet systematic feature extraction remains challenging. This multi‐center study developed and validated an automated prognostic model using deep learning on digitized whole‐slide images from 873 PDAC patients with surgical resection across three academic centers. The CrossFormer architecture achieved superior performance in external validation (area under the curve [AUC] = 0.774), significantly outperforming ResNet‐18 (AUC = 0.716), ResNet‐50 (AUC = 0.737), and DenseNet‐121 (AUC = 0.729). Gradient‐weighted Class Activation Mapping identified key prognostic features including desmoplastic stroma, high nuclear‐to‐cytoplasmic ratio, tumor necrosis, and immune cell infiltration. The pathomics signature effectively stratified patients into low‐risk and high‐risk groups with significant survival differences (*p* < 0.001). Critically, carbohydrate antigen 19‐9 (CA19‐9) retained prognostic value only in low‐risk patients (hazard ratio [HR] = 2.70, *p* < 0.001) but not in high‐risk patients (HR = 0.998, *p* = 0.990). High‐risk patients derived substantial benefit from adjuvant chemotherapy (HR = 0.56, *p* = 0.038), whereas low‐risk patients showed no significant benefit (HR = 0.83, *p* = 0.562). These findings provide actionable clinical insights: treatment intensification for high‐risk patients and CA19‐9‐guided monitoring for low‐risk patients. This validated, interpretable model transforms routine H&E slides into quantitative prognostic tools, enabling personalized treatment strategies without additional testing costs.

## Graphical abstract

This study develops a deep learning‐based pathomics model to predict survival outcomes in pancreatic cancer patients. The CrossFormer architecture analyzes routine H&E‐stained tissue slides, identifying key prognostic features including stromal patterns, cellular characteristics, and immune infiltration. The model successfully stratifies patients into high‐ and low‐risk groups, enabling personalized treatment decisions and demonstrating robust performance across multiple independent clinical cohorts.

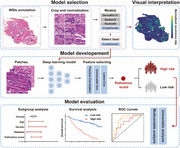

## 1 Introduction

Pancreatic ductal adenocarcinoma (PDAC) remains one of the deadliest malignancies with a 5‐year survival rate about 13%, and is projected to become the second leading cause of cancer‐related deaths by 2040 [1, 2]. The complex molecular landscape of PDAC, characterized by Bailey et al.’s [3] identification of four distinct molecular subtypes (squamous, pancreatic progenitor, immunogenic, and aberrantly differentiated endocrine exocrine) and Collisson et al. [4] classification revealing differential treatment responses between classical and basal‐like subtypes, underlies its therapeutic resistance and clinical heterogeneity. This molecular diversity translates into varied histopathological presentations and treatment outcomes [5, 6], necessitating more sophisticated prognostic tools.

Current prognostic assessment tools demonstrate significant limitations. Serum carbohydrate antigen 19‐9 (CA19‐9), the only Food and Drug Administration (FDA)‐approved biomarker for PDAC, shows false negativity in 5%–10% of Lewis antigen‐negative individuals and lacks specificity in obstructive jaundice [7]. Recent pathomics studies have achieved moderate success, with Chen et al. [8] and Lee et al. [9] reporting a C‐index of 0.653 and 0.726 for survival prediction on The Cancer Genome Atlas data. However, these approaches have not fully captured the complex spatial relationships and morphological heterogeneity inherent in PDAC tissue architecture.

Hematoxylin and eosin (H&E)‐stained slides represent an underutilized resource containing morphological patterns that reflect underlying molecular alterations [5, 6]. Specific histological features—including tumor‐stroma ratio, perineural invasion patterns, and immune cell distribution—have demonstrated independent prognostic value in multiple studies [10, 11, 12]. Manual assessment of these features suffers from inter‐observer variability and cannot integrate multiple morphological patterns into unified risk scores [13]. The spatial heterogeneity of PDAC, where different regions exhibit distinct morphological and molecular characteristics [5, 14], further complicates traditional pathological evaluation.

Deep learning offers a solution by enabling objective, quantitative analysis of complex morphological patterns. Convolutional neural networks (CNNs) have achieved expert‐level performance in various cancer types, with recent transformer architectures demonstrating superior capability in capturing long‐range spatial dependencies. In PDAC specifically, Cao et al. [15] developed algorithms matching contrast‐enhanced CT performance for detection while Davide Placido et al. [16] used deep learning on longitudinal real‐world clinical data to predict the likelihood of PDAC occurrence, offering a potential framework for early detection of this aggressive cancer. However, existing studies primarily focused on diagnostic tasks rather than prognostic stratification and treatment guidance.

This study addresses these critical gaps by developing and validating a comprehensive deep learning‐based pathomics model using 873 PDAC cases with surgical resection across three academic centers. We systematically compared multiple deep learning architectures including conventional CNNs and transformer‐based models to identify optimal feature extraction strategies for PDAC histopathology. Through gradient‐weighted class activation mapping, we sought to reveal interpretable histopathological patterns underlying model predictions, bridging the gap between artificial intelligence and clinical pathology. Furthermore, we investigated how pathomics signatures interact with established prognostic markers like CA19‐9 and evaluated differential treatment benefits based on pathomics risk stratification, ultimately aiming to transform routine H&E slides into actionable tools for personalized PDAC management.

## 2 Results

### 2.1 Patient Characteristics

**FIGURE 1:**
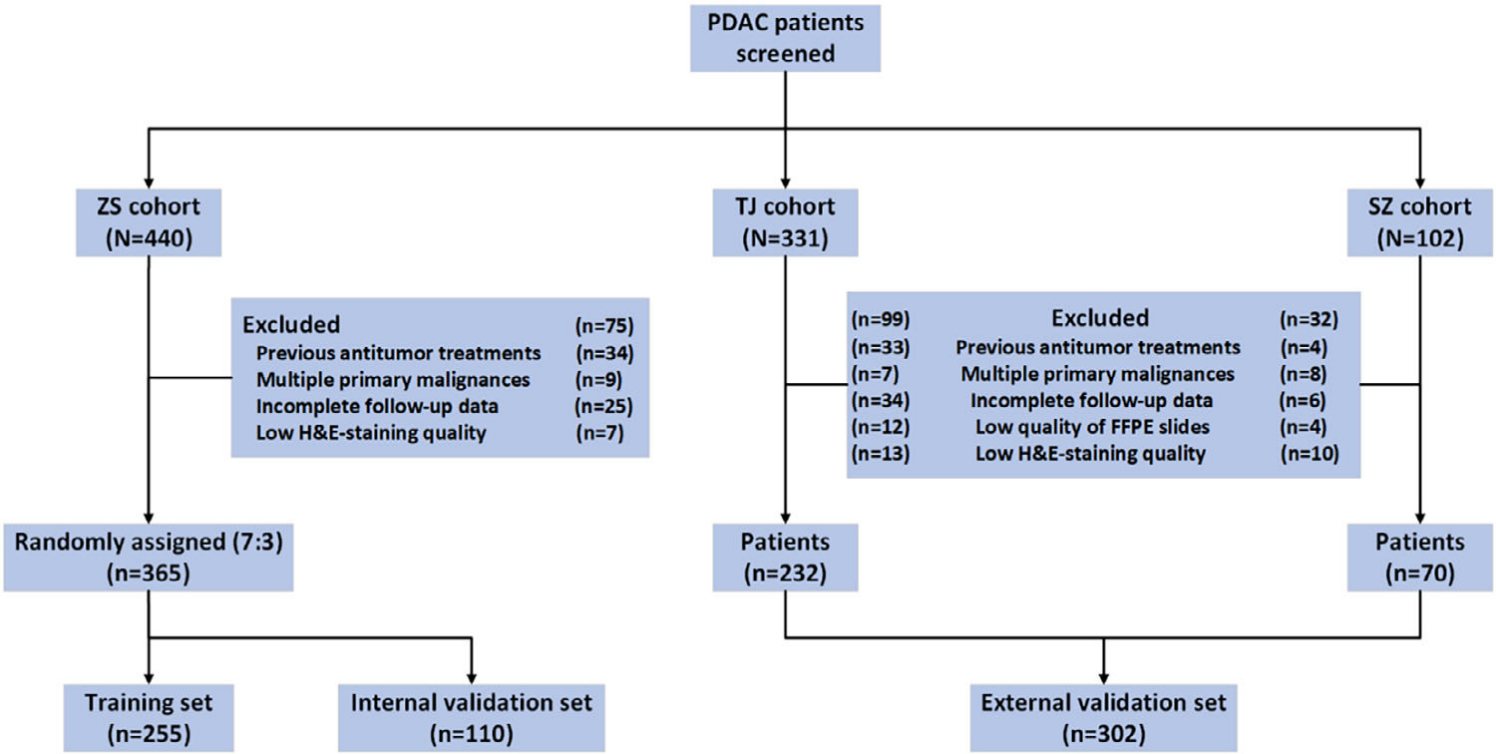
Flow diagram of patient inclusion across the study cohorts. ZS cohort, Zhongshan Hospital cohort, TJ&SZ cohort; Tianjin Medical University Cancer Institute and Hospital and the First Affiliated Hospital of Soochow University cohort.

A total of 440 cases from the Zhongshan Hospital (ZS) cohort, 331 cases from the Tianjin Medical University Cancer Institute and Hospital (TJ) cohort, and 102 cases from the First Affiliated Hospital of Soochow University (SZ) cohort were enrolled in this study. After applying the exclusion criteria, 365 PDAC patients with complete follow‐up and clinicopathological data from the ZS cohort were included in the construction of the prognostic model. These cases were randomly split into training (*n* = 255) and internal validation sets (*n* = 110) at a 7:3 ratio. Additionally, 302 PDAC patients from the TJ&SZ cohort, with the same required data, were included in the external validation set (Figure 1).

The baseline characteristics of the cohorts were summarized in Table 1. Inter‐cohort comparisons revealed significant differences in age, surgery, differentiation, T stage, adjuvant chemotherapy, and CA19‐9 levels, while other clinicopathological parameters were similar across groups. These differences likely reflect underlying variations in patient demographics, institutional treatment practices, and stage distribution across the participating centers. Notably, the external validation cohort demonstrated significantly worse survival outcomes, with a median overall survival (OS) of 24.1 months, compared to 48.1 months in the training cohort (*p* < 0.001) and 35.2 months in the internal validation cohort (*p* < 0.001, Figure S1A). A similar trend was observed for recurrence‐free survival (RFS), with the external validation cohort showing 12.2 months versus 20.1 months in the training cohort (*p* < 0.001) and 19.1 months in the internal validation cohort (*p* < 0.001, Figure S1B).

**TABLE 1:** Clinicopathological characteristics of patients in the three independent cohorts.

	Training set	Internal validation set	External validation set	
Variables	(*n* = 255)	(*n* = 110)	(*n* = 302)	*p*‐value
Gender				0.287
Female	118 (46.3%)	44 (40.0%)	121 (40.1%)	
Male	137 (53.7%)	66 (60.0%)	181 (59.9%)	
Age				0.007
<65	117 (45.9%)	51 (46.4%)	176 (58.3%)	
≥65	138 (54.1%)	59 (53.6%)	126 (41.7%)	
Surgery				<0.001
DP	125 (49.0%)	50 (45.5%)	85 (28.1%)	
PD	122 (47.9%)	58 (52.7%)	217 (71.9%)	
TP	8 (3.1%)	2 (1.8%)	0 (0%)	
Differentiation				0.036
I‐II	144 (56.5%)	64 (58.2%)	142 (47.0%)	
III	111 (43.5%)	46 (41.8%)	160 (53.0%)	
T stage				<0.001
T1	77 (30.2%)	37 (33.6%)	36 (12.0%)	
T2	130 (51.0%)	59 (53.6%)	203 (67.2%)	
T3‐4	48 (18.8%)	14 (12.7%)	63 (20.8%)	
N stage				0.683
N0	154 (60.4%)	63 (57.3%)	184 60.9%)	
N1	85 (33.3%)	43 (39.1%)	99 (32.8%)	0.106
N2	16 (6.3%)	4 (3.6%)	19 (6.3%)	
M stage	246 (96.5%)	108 (98.2%)	299 (99.0%)	
M0	9 (3.5%)	2 (1.8%)	3 (1.0%)	
M1				
TNM stage				0.609
I	125 (49.0%)	56 (50.9%)	147 (48.7%)	
II	104 (40.8%)	45 (40.9%)	132 (43.7%)	
III	17 (6.7%)	7 (6.4%)	20 (6.6%)	
IV	9 (3.5%)	2 (1.8%)	3 (1.0%)	
Adjuvant chemotherapy				<0.001
No	34 (13.3%)	21 (19.1%)	88 (29.1%)	
Yes	221 (86.7%)	89 (80.9%)	214 (70.9%)	
Diabetes				0.101
No	182 (71.4%)	71 (64.5%)	227 (75.2%)	
Yes	73 (28.6%)	39 (35.5%)	75 (24.8%)	
Hypertension				0.109
No	149 (58.4%)	65 (59.1%)	201 (66.6%)	
Yes	106 (41.6%)	45 (40.9%)	101 (33.4%)	
Microvascular invasion				0.477
No	184 (72.2%)	86 (78.2%)	225 (74.5%)	
Yes	71 (27.8%)	24 (21.8%)	77 (25.5%)	
Perineural invasion				0.394
No	54 (21.2%)	20 (18.2%)	73 (24.2%)	
Yes	201 (78.8%)	90 (81.8%)	229 (75.8%)	
CA19‐9				<0.001
<200	176 (69.0%)	76 (69.1%)	158 (52.3%)	
≥200	79 (31.0%)	34 (30.9%)	144 (47.7%)	

Abbreviations: DP, distal pancreatectomy; PD, pancreaticoduodenectomy; TP, total pancreatectomy.

### 2.2 Performance of the Deep Learning Models

**FIGURE 2:**
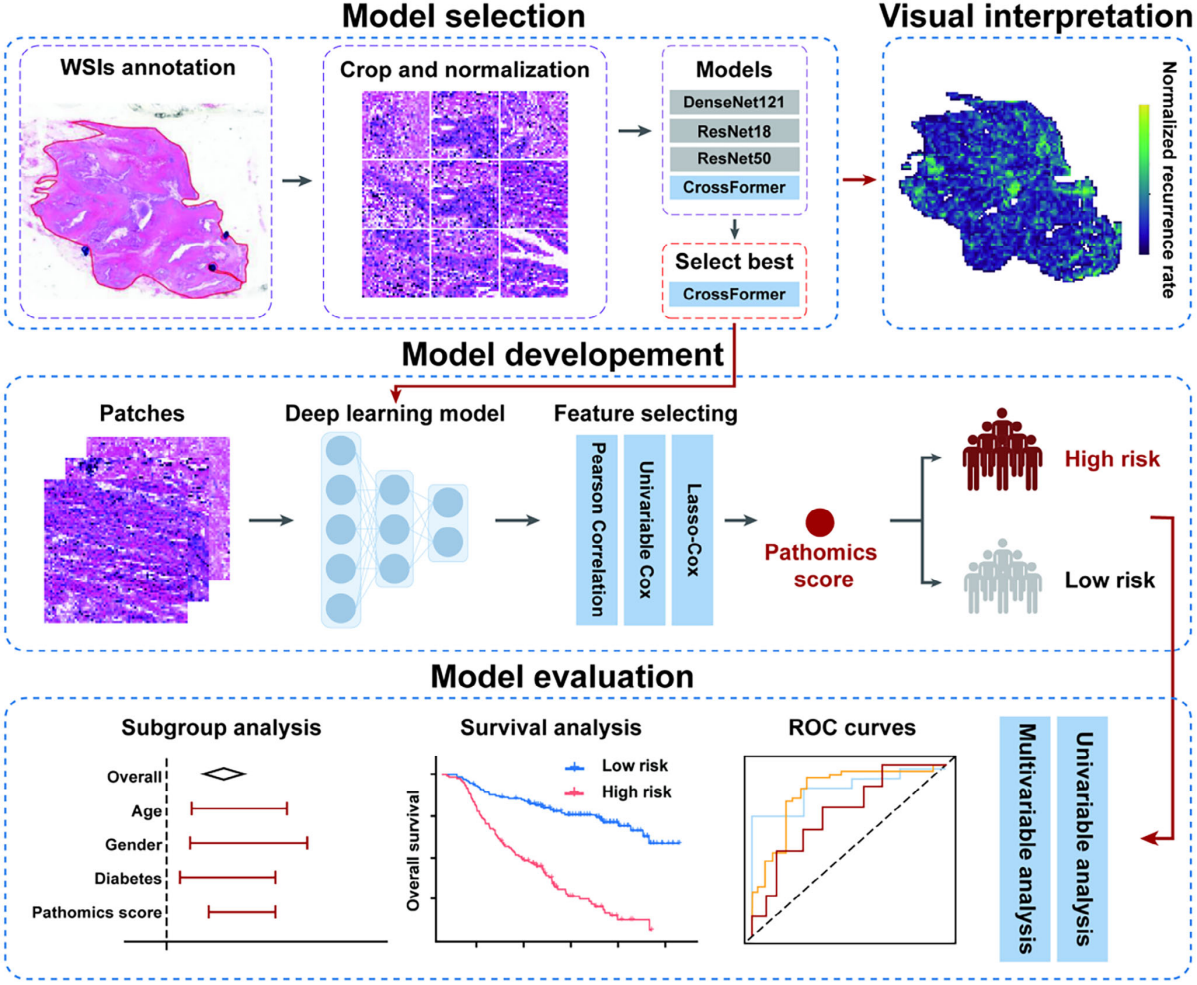
Overview of pathomics prognostic model selection, construction and evaluation. The workflow comprised four main stages: Model Selection: Whole slide images (WSIs) underwent tumor region annotation, followed by segmentation and color normalization. Four deep learning architectures were evaluated, with the CrossFormer model demonstrating optimal performance and subsequently selected for model development. Visual Interpretation: Grad‐CAM was implemented to visualize and interpret the model's decision‐making process. Model Development: Processed image patches were used to train the deep learning model. Extracted prognostic features underwent systematic three‐tier filtration. The final output generated a quantitative pathomics score for patient risk stratification into high‐ and low‐risk groups. Model Evaluation: Comprehensive validation included univariable and multivariable analyses, Receiver Operating Characteristic curves, and additional statistical methods to assess model accuracy and robustness in predicting overall survival and recurrence‐free survival.

To provide a comprehensive overview of our analytical approach, we present the complete workflow of pathomics model development and validation (Figure 2).

**FIGURE 3:**
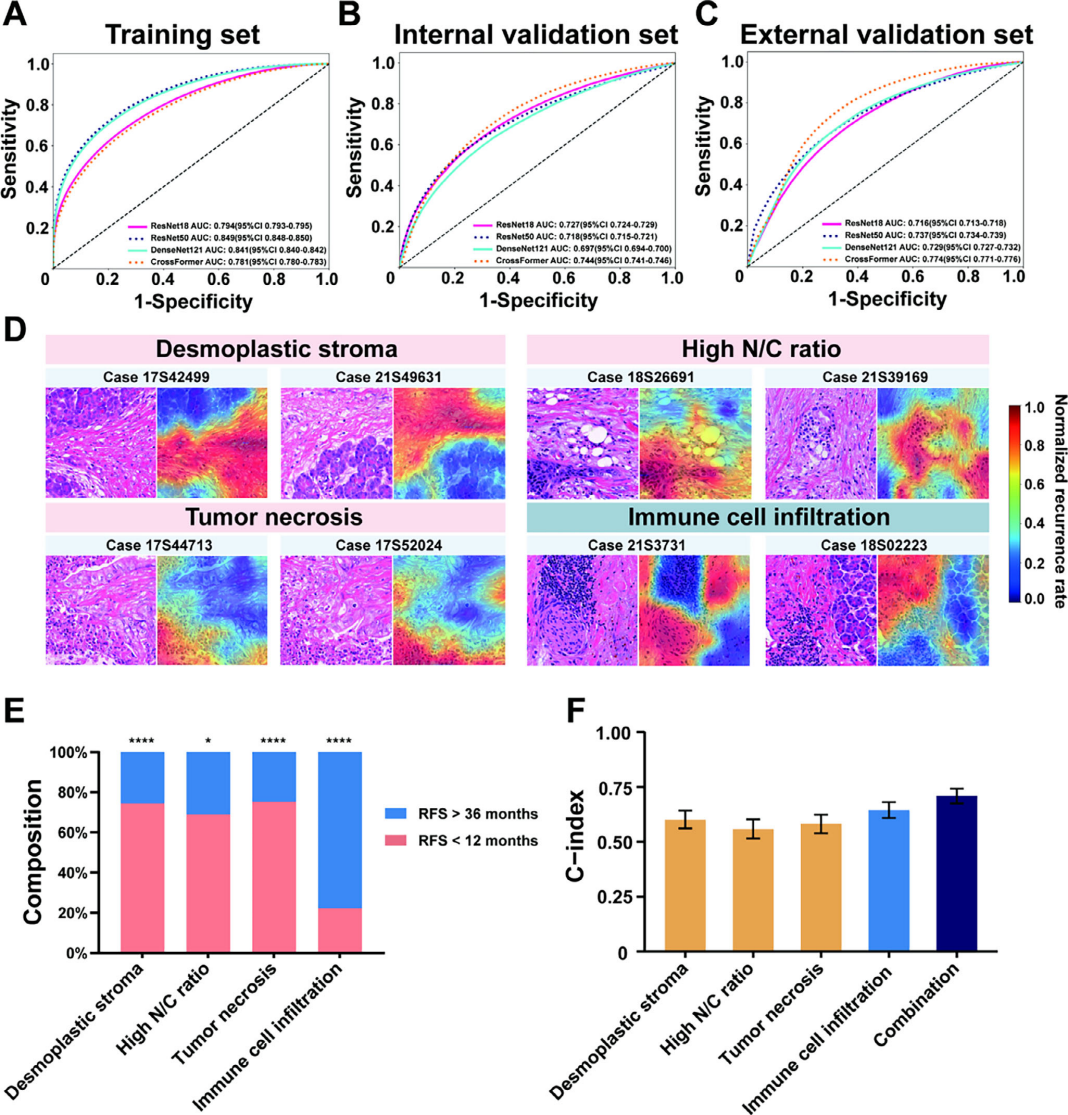
Model performance comparison and feature visualization analysis. (A–C) ROC curves comparing the performance of ResNet18, ResNet50, DenseNet121, and CrossFormer architectures across training, internal validation, and external validation sets. (D) Grad‐CAM generated heatmaps highlighting regions: high recurrence risk features (desmoplastic stroma, high nuclear‐to‐cytoplasmic(N/C) ratio and tumor necrosis) and low recurrence risk features (immune cell infiltration). Original magnification, ×200. (E) Stacked histogram depicting the distribution of identified pathological features stratified by recurrence‐free survival. (F) C‐index for individual features and their combined predictive performance; ^*^, *p* < 0.05; ^***^; *p* < 0.001, N/C, nuclear‐to‐cytoplasmic.

A comparative analysis of deep learning architectures revealed that although the CrossFormer model demonstrated modest performance in the training set (area under the curve [AUC]: 0.781, 95% confidence interval [CI]: 0.780–0.783, Figure 3A), it outperformed traditional models in predicting WSI‐level outcomes. It achieved the highest AUC of 0.744 (95% CI: 0.741–0.746) on the internal validation set, significantly surpassing other architectures, including ResNet18 (AUC: 0.727, 95% CI: 0.724–0.729), ResNet50 (AUC: 0.718, 95% CI: 0.715–0.721), and DenseNet121 (AUC: 0.697, 95% CI: 0.694–0.700, Figure 3B). This superior performance was further validated on the external validation set, where CrossFormer maintained its leading position with an AUC of 0.774 (95% CI: 0.771–0.776), compared to ResNet18 (0.716, 95% CI: 0.713–0.718), ResNet50 (0.737, 95% CI: 0.734–0.739) and DenseNet121 (AUC: 0.729, 95% CI: 0.727–0.732, Figure 3C). A detailed analysis of the external validation set revealed balanced performance across multiple metrics: sensitivity of 0.769, specificity of 0.664, positive predictive value (PPV) of 0.879, and negative predictive value (NPV) of 0.476 (Table S1). Given these comprehensive results and the model's consistent performance across all datasets—especially its strong performance on the validation set—we selected the CrossFormer model for feature aggregation in our multi‐instance learning framework. Its robust generalizability and balanced metrics make it particularly suitable for this pathological imaging analysis.

### 2.3 Visual Interpretation and Validation Through Grad‐CAM Analysis

To enhance the interpretability of the deep learning model's decision‐making, we used Gradient‐weighted Class Activation Mapping (Grad‐CAM). The resulting heatmaps highlighted regions in the final convolutional layer that significantly influenced cancer classification outcomes. Expert pathologists identified four key pathological features with strong predictive value (Figure 3D). Deep red regions in the heatmap corresponded to desmoplastic stroma, a high nuclear‐to‐cytoplasmic (N/C) ratio, and tumor necrosis, all of which were strongly associated with poor prognosis. In contrast, deep blue regions, representing immune cell infiltration, were linked to a more favorable prognosis.

To validate the prognostic significance of these identified features, we analyzed the proportion of patients with different outcomes for each pathological characteristic. For the three features associated with poor prognosis, more than 60% of patients experienced adverse outcomes. In contrast, 72% of patients with prominent immune cell infiltration demonstrated favorable survival (Figure 3E). The prognostic robustness of the selected features was further confirmed using Harrell's C‐indices in the training cohort. Among individual features, immune cell infiltration demonstrated the highest discriminative power (C‐index: 0.646; 95% CI: 0.611–0.681), followed by dense desmoplastic stroma (C‐index: 0.602; 95% CI: 0.559–0.640), tumor necrosis (C‐index: 0.581; 95% CI: 0.537–0.623), and high nuclear‐to‐cytoplasmic ratio (C‐index: 0.558; 95% CI: 0.514–0.599). Each of these features exceeded the 0.5 threshold, indicating meaningful prognostic relevance. Importantly, when integrated into a composite pathomics signature, the combined model yielded a significantly higher C‐index of 0.708 (95% CI: 0.673–0.742), underscoring the added prognostic value of multi‐feature integration compared to individual assessments alone (Figure 3F).

### 2.4 Prognostic Value of Pathomics Signature

**FIGURE 4:**
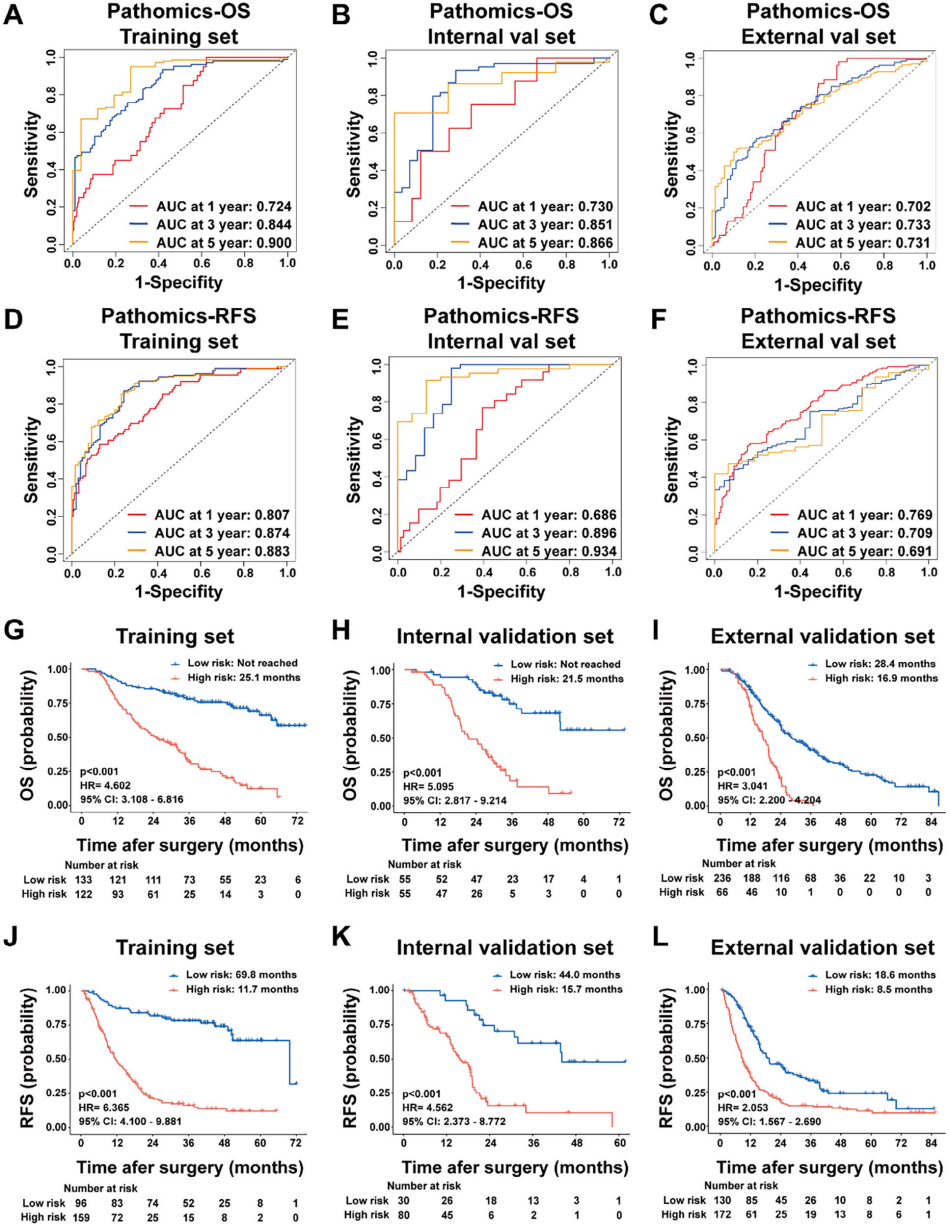
Prognostic performance of pathomics signatures for OS and RFS. (A–C) ROC curves demonstrating the predictive accuracy of pathomics signature for 1‐, 3‐, and 5‐year OS across training, internal validation, and external validation cohorts. (D–F) ROC curves illustrating the predictive performance of pathomics signature for 1‐, 2‐, and 3‐year RFS across training, internal validation, and external validation cohorts. (G–I) Kaplan–Meier analysis of OS based on pathomics signature risk stratification in training, internal validation, and external validation cohorts. (J–L) Kaplan–Meier analysis of RFS based on pathomics signature risk stratification in training, internal validation, and external validation cohorts. OS, overall survival; RFS, recurrence‐free survival; HR, hazard ratio; CI, confidence interval.

This signature was constructed by integrating deep learning‐derived features from WSIs, followed by a sequential three‐tier feature selection process: correlation‐based filtering, univariate Cox regression, and Least Absolute Shrinkage and Selection Operator (LASSO)‐Cox modeling. The final model incorporated patch‐level predictive outputs and risk activation mapping data from the training cohort. The resulting algorithms, termed Pathomics‐OS and Pathomics‐RFS, provided quantitative risk assessments for individual patients across all cohorts. Pathomics‐OS demonstrated robust prognostic performance in the training set, with AUC values of 0.724, 0.844, and 0.900 for 1‐, 3‐, and 5‐year OS, respectively (Figure 4A). The model maintained high discriminative capability in the internal validation cohort, with AUC values of 0.730, 0.851, and 0.866 (Figure 4B). External validation revealed moderate to strong performance, with AUC values of 0.702, 0.733, and 0.731 for the same time points (Figure 4C). Similar performance was observed for the Pathomics‐RFS model (Figure 4D–F).

To evaluate the clinical utility of our pathomics signatures, we conducted comprehensive comparisons with established prognostic factors, including Tumor‐Node‐Metastasis (TNM) staging and CA19‐9 levels (Table S2). Across all validation cohorts, the pathomics signatures consistently demonstrated superior discriminative performance. At every evaluated time point, pathomics signatures achieved higher AUC values for both OS and RFS compared to TNM staging and CA19‐9, highlighting their sustained temporal advantage in prognostic accuracy. In the training cohort, the pathomics signatures outperformed traditional markers with C‐indices of 0.752 for RFS and 0.742 for OS, markedly exceeding those of TNM staging (0.594 and 0.585, respectively) and CA19‐9 (0.562 and 0.555, respectively). This superior performance was retained in the external validation cohort, where pathomics‐RFS and pathomics‐OS achieved C‐indices of 0.673 and 0.651, respectively—again outperforming TNM staging (0.553 and 0.552) and CA19‐9 (0.568 and 0.551). These results underscore the strong generalizability and enhanced prognostic capability of the pathomics approach across diverse clinical settings.

Risk stratification thresholds were determined using X‐tile analysis, yielding cutoff values of 3.431 for Pathomics‐OS and 3.093 for Pathomics‐RFS in the training cohort. These thresholds were applied to stratify patients into high‐ and low‐risk groups across all cohorts, and the lower pathomics signature scores represented the higher prognostic risk. Survival analyses revealed significantly worse outcomes in high‐risk patients for both OS (training: *p* < 0.001, hazard ratio [HR] = 4.602, 95% CI: 3.108–6.816, Figure 4G; internal validation: *p* < 0.001, HR = 5.095, 95% CI: 2.817–9.214, Figure 4H; external validation: *p* < 0.001, HR = 3.041, 95% CI: 2.200–4.204, Figure 4I) and RFS (training: *p* < 0.001, HR = 6.365, 95% CI: 4.100–9.881, Figure 4J; internal validation: *p* < 0.001, HR = 4.562, 95% CI: 2.373–8.772, Figure 4K; external validation: *p* < 0.001, HR = 2.053, 95% CI: 1.567–2.690, Figure 4L). Multivariate Cox regression analyses, incorporating clinicopathological factors identified in univariate analyses (*p* < 0.05), confirmed both signatures as independent prognostic indicators (Table 2; Tables S3–S5). Importantly, the pathomics signatures retained strong independent prognostic value even after adjustment for established clinical variables, including lymph node status and other conventional prognostic factors. Pathomics‐OS retained its prognostic significance across cohorts (training: *p* < 0.001, HR = 4.494, 95% CI: 2.988–6.760; internal validation: *p* < 0.001, HR = 4.011, 95% CI: 2.084–7.720; external validation: *p* < 0.001, HR = 2.763, 95% CI: 1.974–3.868). Similarly, Pathomics‐RFS demonstrated robust independent prognostic value (training: *p* < 0.001, HR = 5.293, 95% CI: 3.365–8.325; internal validation: *p* < 0.001, HR = 4.05, 95% CI: 2.058–7.980; external validation: *p* < 0.001, HR = 1.939, 95% CI: 1.476–2.546). These results confirm the robustness of our deep learning framework for PDAC prognostic stratification.

**TABLE 2:** Univariate and multivariate Cox regression analyses of the pathomics signature in relation to overall survival and recurrence‐free survival.

		Overall survival			Recurrence‐free survival		
Sets	Variables	Univariate *p*‐value	Multivariate *p*‐value	Multivariate HR (95% CI)	Univariate *p*‐value	Multivariate *p*‐value	Multivariate HR (95% CI)
Training set	Pathomics‐OS						
	Low‐risk / High‐risk	<0.001	<0.001	4.494 (2.988 to 6.760)			
	Pathomics‐RFS						
	Low‐risk / High‐risk				<0.001	<0.001	5.293 (3.365 to 8.325)
Internal validation set	Pathomics‐OS						
	Low‐risk / High‐risk	<0.001	<0.001	4.011 (2.084 to 7.720)			
	Pathomics‐RFS						
	Low‐risk / High‐risk				<0.001	<0.001	4.053 (2.058 to 7.980)
External validation set	Pathomics‐OS						
	Low‐risk / High‐risk	<0.001	<0.001	2.763 (1.974 to 3.868)			
	Pathomics‐RFS						
	Low‐risk / High‐risk				<0.001	<0.001	1.939 (1.476 to 2.546)

To further evaluate the robustness and generalizability of the pathomics signatures, we performed mixed‐effects Cox regression modeling with study center included as a random effect. The model yielded a center‐specific random effect variance of 0.325, indicating moderate variability between institutions while preserving a strong overall prognostic signal for the pathomics signature (*p* < 0.001, HR = 3.729, 95% CI: 2.993–4.645). Complementary meta‐analytic assessment across institutions revealed moderate heterogeneity (I^2^ = 46.5%), though this did not reach statistical significance (Cochran's Q‐test *p* = 0.159). The pooled effect estimate remained significant (*p* < 0.001, HR = 1.372, 95% CI: 1.041–1.703), supporting the consistent prognostic value of the pathomics signature across diverse clinical settings. Subsequently, subgroup analyses demonstrated that the Pathomics‐OS signature maintained consistent prognostic value across a range of patient characteristics, with the exception of those with stage III–IV disease. This exception was likely attributable to the limited number of late‐stage cases in the ZS cohort (Figure S2). Similar trends were observed for the Pathomics‐RFS signature (Figure S3). These findings were further corroborated in the external validation cohort, where both Pathomics‐OS (Figure S4) and Pathomics‐RFS (Figure S5) showed sustained prognostic performance, reinforcing the predictive strength and generalizability of the model across independent clinical populations.

### 2.5 Prognostic Variations of Serum CA19‐9 Levels Intercepted by Pathomics Signature

**FIGURE 5:**
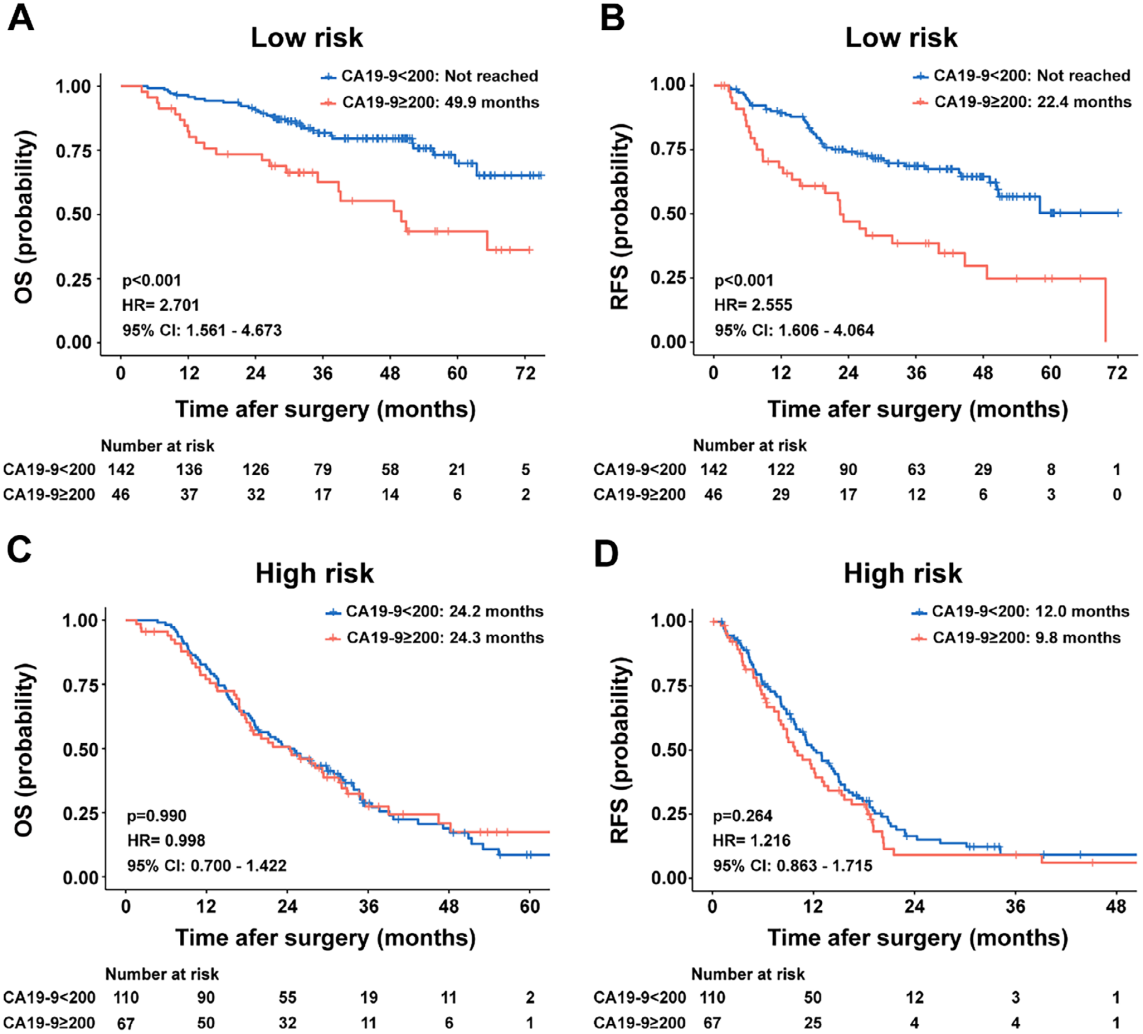
The prognostic value of CA19‐9 intercepted by the pathomics signature. (A, B) Kaplan–Meier analyses of OS and RFS stratified by serum CA19‐9 levels in the low‐risk pathomics signature group. (C, D) Kaplan‐Meier analyses of OS and RFS stratified by serum CA19‐9 levels in the high‐risk pathomics signature group. OS, overall survival; RFS, recurrence‐free survival; HR, hazard ratio; CI, confidence interval.

CA19‐9 is the only FDA‐approved biomarker for PDAC, playing a key role in diagnosis, tumor burden assessment, and monitoring therapeutic response [17, 18]. While its prognostic significance has been well established in pre‐ and peri‐operative settings, our interaction analyses revealed that the pathomics signature significantly modulates CA19‐9′s prognostic utility. Specifically, in the low‐risk group, CA19‐9 demonstrated strong predictive value. Patients with low CA19‐9 levels had significantly better median OS compared to those with high levels (not reached vs 49.9 months, *p* < 0.001, HR = 2.701, 95% Cl: 1.561–4.673, Figure 5A) and similarly better RFS (not reached vs 22.4 months, *p* < 0.001, HR = 2.555, 95% Cl: 1.606–4.064, Figure 5B). However, in the high‐risk group, CA19‐9 levels did not significantly correlate with prognosis (OS: 24.2 vs 24.3 months, *p* = 0.998, Figure 5C; RFS: 12.0 vs 9.8 months, *p* = 0.264, Figure 5D). These findings suggest that the pathomics signature plays a crucial role in modulating the prognostic accuracy of CA19‐9.

### 2.6 Therapeutic Response to Adjuvant Chemotherapy Based on Pathomics Signature

To assess the clinical applicability of the pathomics signature in guiding treatment decisions, we first examined its associations with established clinicopathological characteristics. The high‐risk pathomics group showed significant associations with aggressive tumor features including differentiation, perineural invasion, T stage, N stage, and CA19‐9 levels (Table S6), indicating that our model captured biologically relevant aggressive features. Then, we evaluated the impact of adjuvant chemotherapy within stratified pathomics risk groups. Since RFS was usually utilized for predicting the response to adjuvant chemotherapy, the survival analysis showed that patients receiving adjuvant chemotherapy in the high‐risk group experienced significantly delayed recurrences, with longer RFS (*p* = 0.038, HR = 0.563, 95% Cl: 0.327–0.969, Figure S6A) compared to those who did not receive adjuvant therapy. In contrast, among patients in the low‐risk group, adjuvant chemotherapy did not confer a significant RFS advantage (*p* = 0.562, HR = 0.827, 95% Cl: 0.434–1.574, Figure S6B). To determine whether this predictive capability was unique to the pathomics signature, we further analyzed CA19‐9 as a comparator biomarker using an RFS‐optimized cutoff value. Notably, CA19‐9 failed to identify patients who would benefit from adjuvant chemotherapy: neither the CA19‐9 high group (HR = 0.729, 95% CI: 0.440–1.209, *p* = 0.221, Figure S6C) nor the CA19‐9 low group (HR = 0.989, 95% CI: 0.486–2.012, *p* = 0.976, Figure S6D) showed significant improvement with adjuvant therapy. These findings highlight the potential of pathomics signatures to serve as predictive biomarkers for tailoring adjuvant chemotherapy in PDAC. High‐risk patients appear to derive meaningful benefit from adjuvant treatment, supporting a more personalized approach to postoperative therapeutic decision‐making.

## 3 Discussion

Histopathological H&E slides encode comprehensive information about tumor biology, including cellular morphology, stromal architecture, and microenvironmental composition [5, 6, 14]. However, traditional pathological assessment faces inherent limitations: subjective interpretation, focus on isolated features, and inability to quantify subtle variations across extensive tissue areas [19]. The integration of digital pathology with deep learning enables systematic, objective quantification of complex histological features [20].

This multi‐center study demonstrates that deep learning‐based pathomics analysis of routine H&E slides provides robust prognostic stratification for PDAC patients by implementing the CrossFormer architecture, a vision transformer employing cross‐scale attention mechanisms to capture long‐range spatial dependencies [21]. Unlike conventional CNNs (ResNet, DenseNet) with limited receptive fields, CrossFormer directly models relationships across tissue sections—critical for PDAC where prognostic features manifest across multiple spatial scales [22, 23]. The CrossFormer architecture's superior performance (AUC = 0.774) over conventional CNNs validates the importance of capturing long‐range spatial dependencies in PDAC histopathology, where tumor‐stroma interactions and immune infiltration patterns extend across tissue regions [24, 25, 26]. Our model's ability to maintain performance across independent cohorts suggests successful learning of generalizable biological features rather than institution‐specific artifacts.

To enhance interpretability, we employed Grad‐CAM visualization, identifying four morphological features consistently associated with poor prognosis: desmoplastic stromal reaction, elevated nuclear‐to‐cytoplasmic ratio, tumor necrosis, and immune cell infiltration patterns. These findings align with established biological mechanisms. Desmoplastic stroma, comprising up to 90% of tumor volume in PDAC, creates a hypoxic microenvironment that promotes chemoresistance through impaired drug delivery and activation of survival pathways [27, 28]. The dense extracellular matrix acts as a physical barrier, with studies showing that hyaluronidase‐mediated stromal depletion enhances gemcitabine delivery and survival in preclinical models [29]. High nuclear‐to‐cytoplasmic ratio reflects increased proliferative activity and chromosomal instability in PDAC [30]. Tumor necrosis indicates rapid growth outpacing vascular supply, releasing damage‐associated molecular patterns that paradoxically promote tumor progression through inflammatory cascades [31, 32, 33, 34]. Immune infiltration patterns, particularly the balance between cytotoxic T cells and immunosuppressive macrophages, determine the tumor's immune contexture and treatment responsiveness [35, 36]. Critically, our model captures the integrated prognostic impact of these features within a unified quantitative framework rather than detecting them in isolation. The concordance between model‐identified features and established mechanisms strengthens biological plausibility and clinical interpretability.

Our finding that CA19‐9 loses prognostic value in pathomics‐defined high‐risk patients while maintaining significance in low‐risk groups reveals important biological insights. High‐risk tumors identified by our model likely represent terminally dedifferentiated phenotypes with multiple adverse features—dense desmoplasia, high cellular atypia, extensive necrosis—that supersede the prognostic information from CA19‐9 alone. The uniform elevation of CA19‐9 in high‐risk patients creates a ceiling effect, eliminating its discriminatory power. Conversely, in low‐risk patients with more differentiated histology, CA19‐9 variations reflect tumor burden and sialylated Lewis antigen production capacity [17, 37], providing additional prognostic information. This differential utility suggests that morphological features visible in H&E slides capture more fundamental aspects of tumor biology than serum markers in advanced disease states.

The differential chemotherapy benefit observed between risk groups has immediate clinical implications. High‐risk patients' substantial benefit from adjuvant therapy likely reflects their aggressive tumor biology requiring systemic treatment to control micrometastatic disease. These tumors may harbor higher mutational burdens and proliferative indices, making them more chemosensitive despite worse baseline prognosis [38, 39, 40, 41]. Our pathomics signature offers several advantages over existing prognostic tools. Unlike molecular profiling requiring fresh tissue and specialized platforms [42], our approach leverages universally available H&E slides without additional costs. The model processes entire tumor sections rather than selected regions, capturing intratumoral heterogeneity that single‐biopsy molecular assays miss [20]. The continuous risk score provides more nuanced stratification than binary classifications, enabling personalized risk assessment along a spectrum rather than arbitrary cutoffs.

The translational value of our pathomics signature extends to immediate clinical applicability. First, it leverages universally available H&E‐stained slides without requiring additional molecular testing, specialized equipment, or increased costs—making it immediately implementable in routine clinical workflows worldwide. The clinical applications are straightforward and actionable: upon surgical resection, pathologists can apply our pathomics algorithm to routine H&E slides, generating a quantitative risk score that stratifies patients into high‐risk and low‐risk groups within hours of specimen processing. High‐risk patients, who demonstrated substantial benefit from adjuvant chemotherapy, should be prioritized for aggressive adjuvant treatment regimens, while low‐risk patients may be considered for less intensive approaches or clinical trial enrollment.

Despite these advancements, several limitations warrant careful consideration when interpreting our findings. First, our model was trained on a single‐center cohort (ZS), which, while ensuring rigorous external validation, may not fully capture the institutional heterogeneity present in broader clinical settings. Although our external validation across two independent centers demonstrated robust generalizability, future studies employing multi‐center training with domain adaptation techniques may further enhance model performance. Second, the retrospective design inherently introduces potential selection bias and confounding factors that may influence outcomes. To maintain cohort homogeneity and isolate the intrinsic prognostic value of tumor morphology, we specifically excluded patients who had received prior anti‐tumor therapies, including neoadjuvant treatment. While this approach allowed us to establish a clear baseline for evaluating the prognostic utility of the pathomics signature in treatment‐naïve tumors, it limits generalizability to the broader PDAC population, particularly as neoadjuvant therapy becomes increasingly integrated into standard clinical practice [43]. Third, focusing exclusively on primary tumor tissue may overlook prognostic information in nodal microenvironments. Fourth, despite our multi‐center cohort of 873 patients, relatively few advanced‐stage cases limit conclusions for this subgroup. To address these limitations, we are actively pursuing several initiatives. We are establishing a prospective, multi‐center consortium involving additional academic medical centers to collect standardized H&E whole‐slide images with harmonized clinical annotations. We plan to implement federated learning approaches that enable model training across multiple institutions while preserving data privacy and maintaining external validation integrity. We are also initiating complementary studies specifically focused on neoadjuvant‐treated patients to develop treatment response prediction models using paired pre‐ and post‐treatment specimens. Additionally, integrating multi‐omics data—genomic alterations (KRAS, TP53, SMAD4), transcriptomic subtypes, and proteomic profiles—holds promise for comprehensive prognostic frameworks advancing PDAC precision oncology.

## 4 Conclusion

In summary, we developed a deep learning–based pathomics model transforming routine H&E slides into quantitative prognostic tools for PDAC. The model demonstrated consistent multi‐institutional performance, identified biologically validated prognostic features through interpretable techniques, revealed context‐dependent CA19‐9 utility, and successfully predicted adjuvant chemotherapy benefit. As a scalable, cost‐effective approach requiring no specialized molecular testing, our pathomics framework represents a practical step toward individualized, data‐driven PDAC management.

## 5 Experimental Section

### 5.1 Ethics Statement and Patient Cohorts

This retrospective study analyzed anonymized digital images from PDAC tissue samples, and was conducted in accordance with the Declaration of Helsinki, with approval from the Ethics Committee of Zhongshan Hospital, Fudan University (B2025‐207R), Tianjin Medical University Cancer Institute and Hospital (EK20240007), and the First Affiliated Hospital of Soochow University (EC2025036). We enrolled three independent cohorts: ZS cohort (*n* = 440) from Zhongshan Hospital, Fudan University (September 2017‐December 2021), TJ cohort (*n* = 331) from Tianjin Medical University Cancer Institute and Hospital (January 2016‐December 2021), and SZ cohort (*n* = 102) from the First Affiliated Hospital of Soochow University (January 2016‐January 2022). This multi‐center design was intentionally adopted to enhance the generalizability of the findings and to evaluate model performance across varied patient populations, clinical practices, and institutional workflows.

Inclusion criteria were: (1) histologically confirmed PDAC with no distal metastasis; (2) complete clinicopathological data; (3) availability of high‐quality, digitized H&E‐stained slides from surgical resection specimens; and (4) complete follow‐up data for survival analysis. To maintain cohort homogeneity and isolate the intrinsic prognostic value of tumor morphology, patients who had received prior anti‐tumor treatments—including neoadjuvant chemotherapy, radiotherapy, or targeted therapy—were excluded. Additional exclusion criteria included: (1) concurrent malignancies; (2) insufficient tumor tissue for digital analysis; and (3) loss to follow‐up within 30 days after surgery. Collected clinical variables included demographic characteristics, tumor markers (serum CA19‐9), surgical details, pathological features (differentiation, perineural and microvascular invasion), TNM staging (American Joint Committee on Cancer 8th Edition), comorbidities (diabetes, hypertension), receipt of adjuvant chemotherapy, and survival outcomes.

For model development and validation, the ZS cohort was randomly divided into training and internal validation sets in a 7:3 ratio. Model optimization and hyperparameter tuning were conducted on the training set, with model selection based on performance metrics from the validation set. The TJ and SZ cohorts were combined into a unified external validation dataset to evaluate model generalizability. This combination was strategically implemented for two key reasons: First, statistical power considerations—the SZ cohort alone (*n* = 70) would be severely underpowered for reliable external validation, producing unacceptably wide confidence intervals that would preclude meaningful performance assessment. Second, maintaining external validation integrity—combining both TJ and SZ as completely independent external datasets ensures rigorous testing of real‐world generalizability across different institutions, equipment, and protocols.

### 5.2 Survival Follow‐Up

All patients were regularly followed up after surgery, as previously described [44]. The overall survival (OS) was calculated from the date of surgery until death from any cause or the last follow‐up (December 2024). Recurrence‐free survival (RFS) was defined as the time between surgery and either disease recurrence, death, or the last follow‐up, whichever occurred first.

### 5.3 Digital Pathological Image Acquisition and Processing

All tissue specimens were processed according to standardized protocols across participating institutions using formalin‐fixed, paraffin‐embedded (FFPE) tissue blocks sectioned and stained with standard H&E protocols. Analysis focused exclusively on slides containing primary tumor tissue to capture intrinsic histological features, with lymph node specimens intentionally excluded to preserve focus on tumor‐intrinsic morphology and ensure alignment with conventional clinicopathological staging metrics.

The histopathological assessment followed a two‐phase review process. Initially, two board‐certified pathologists independently reviewed all H&E‐stained slides via microscopy, blinded to all clinical and prognostic data. Slides with fragmentation, overlapping, inadequate tissue representation, or contamination were reprocessed and reexamined. WSIs were then captured using a brightfield scanning system (SQF‐120Pro, Shenzhen Shengqiang Technology Co, Ltd.) at 400× magnification, stored in .svs format and processed using QuPath‐0.4.3 software.

Tumor regions of interest (ROIs) were independently annotated by two expert pathologists (A and B), with any discrepancies resolved by a chief pathologist (C), whose assessment was considered final. All annotations underwent secondary review to ensure consistency.

The digital image processing pipeline involved several steps. WSIs were segmented into 512 × 512 pixel tiles at 20 × magnification to facilitate analysis of these high‐dimensional images. Non‐informative tiles, predominantly containing white background pixels, were excluded. Image preprocessing incorporated RGB channel standardization through Z‐score normalization. The Macenko color normalization method was applied to standardize staining variation across all participating centers, ensuring consistent image quality and feature extraction [45]. Training data underwent online augmentation, incorporating random cropping and bi‐directional flipping to enhance dataset diversity, while test patches received only normalization to maintain consistency. This systematic preprocessing pipeline yielded over 1.2 million informative image patches, with each patient contributing thousands of patches. After quality control and selection, more than 800000 high‐quality patches were retained for model training, effectively enhancing the robustness and diversity of the training dataset. All image processing procedures were executed on the OnekeyAI Platform, utilizing specialized tools such as OKT‐crop_WSI2patch for tile segmentation, OKT‐patch2predict for background removal, and OKT‐patch_normalize for color standardization.

### 5.4 Construction and Evaluation of Deep Learning WSI‐Based Model for Predicting Prognosis

Our deep learning framework utilizes a two‐tiered predictive system, combining patch‐based analysis with multiple instance learning to extract comprehensive features from whole‐slide images. Prognostic criteria were established where patients experiencing recurrence within 12 months were classified as having poor prognosis, while those remaining recurrence‐free for over 36 months were considered to have good prognosis. These outcomes were used for risk stratification, with all image patches from a patient assigned the same risk label. We evaluated several neural network architectures, comparing conventional convolutional neural networks (CNNs) with newer transformer‐based approaches. The term “conventional CNNs” refers to traditional deep learning architectures that rely primarily on convolutional operations with local receptive fields to extract hierarchical features from images. These models, including ResNet18, ResNet50, and DenseNet121 [46, 47], process information through sequential convolutional layers, pooling operations, and fully connected layers, focusing on local feature extraction within limited spatial neighborhoods. While these architectures have achieved remarkable success in medical image analysis, they inherently struggle to capture long‐range dependencies and global contextual relationships in large whole‐slide images due to their localized processing approach. In contrast, we also evaluated the CrossFormer architecture [21], a vision transformer that employs a fundamentally different feature extraction strategy. CrossFormer utilizes cross‐scale attention mechanisms that can directly model relationships between distant regions of the image without relying on sequential local convolutions. This enables more effective capture of global pathological patterns, such as tumor‐stroma interactions, immune infiltration distributions, and spatial heterogeneity, that often manifest across multiple scales and tissue regions To ensure robust performance across diverse cohorts, we employed transfer learning, initializing model parameters with weights pre‐trained on the ImageNet dataset, allowing the model to leverage generalized visual features. To promote better generalization, we used a cosine decay learning rate schedule, defined as:

(1) $$\eta_{t}=\eta_{min}^{i}+\frac{1}{2}\left(\eta_{max}^{i}-\eta_{min}^{i}\right)\left(1+\cos\left(\frac{T_{cur}}{T_{i}}\pi \right)\right)$$

Here, $\eta_{min}^{i}=0$ represents the minimum learning rate, $\eta_{max}^{i}=0.01$ is the maximum learning rate, and *T_i_* =  16 corresponds to the total number of iteration epochs. Training specifications varied by architecture: for conventional CNN models (ResNet‐18, ResNet‐50, DenseNet‐121), the initial learning rate was set to 0.01 and models were trained for 8 epochs; for the CrossFormer model, the initial learning rate was 0.001 and training proceeded for 16 epochs with an early stopping criterion where training was halted if validation loss did not improve for 128 consecutive iterations. Regarding the fine‐tuning strategy, we performed full fine‐tuning for the CrossFormer model, meaning all model layers' parameters were updated during the training process with no layers frozen. Softmax cross‐entropy was used as the loss function for all models.

To identify prognostic features from histopathological images, we adapted the Class Activation Mapping (CAM) approach into a Risk Activation Mapping (RAM) method, generating detailed heatmaps at the tile level. Expert pathologists systematically reviewed these heatmaps and annotated regions with distinctive features. We performed quantitative analysis to assess spatial distribution of these features and evaluated their prognostic significance using Harrell's concordance index (C‐index).

Following model training, a multi‐instance learning approach was applied to analyze WSI [48]. Each slice was analyzed using the deep learning model to derive probabilities and labels, denoted as *Patch_prob_* and *Patch_pred_*, retained to two decimal places. Feature aggregation was accomplished through:

1. Histogram Feature Aggregation: Distinct numbers were treated as “bins” to count occurrences across types. Frequencies of *Patch_prob_* and *Patch_pred_* in each bin were tallied and normalized using min‐max normalization, resulting in *Histo_prob_* and *Histo_pred_*.

2. Bag of Words (BoW) Feature Aggregation: A dictionary was constructed from unique elements in *Patch_prob_* and *Patch_pred_*. Each WSI was represented as a vector noting the frequency of each dictionary element, with Term Frequency‐Inverse Document Frequency (TF‐IDF) transformation applied to emphasize informative features, resulting in BoW representations

3. Feature Early Fusion: We integrated *Histo_prob_*, *Histo_pred_*, *Bow_prob_*, and *Bow_pred_* using a feature concatenation method (⨁), combining these into a single comprehensive feature vector:

(2) $$feature_{fusion}=Histo_{prob}⨁Histo_{pred}⨁Bow_{prob}⨁Bow_{pred}$$

Feature extraction resulted in 206 characteristics, comprising two sets of 101 probability features and two predictive label features, all detailed in Table S7. Feature selection was conducted using a structured three‐step pipeline to address dimensionality reduction, eliminate redundancy, and enhance model performance:

1. Correlation‐based filtering: Features with a Pearson correlation coefficient > 0.9 were considered highly collinear, and redundant features were removed to reduce multicollinearity.

2. Feature refinement via univariate Cox regression analysis (*p* < 0.05) to identify features that individually demonstrate significant associations with survival outcomes.

3. LASSO‐Cox modeling: A LASSO‐Cox proportional hazards model was constructed to perform regularized variable selection. This method applies an L1 penalty to shrink less informative feature coefficients to zero, enabling automated selection of the most predictive subset of features while accounting for their combined effects and interactions.

In the LASSO‐Cox modeling step, continuous measurements were used directly without discretization, allowing the model to capture subtle variations in feature expression and their relationship to patient outcomes. The optimal regularization parameter (λ) was determined through 10‐fold cross‐validation in the training cohort, balancing model complexity with predictive performance. The optimal λ values selected for the final models were: λ = 0.179 for the recurrence‐free survival (RFS) task and λ = 0.022 for the overall survival (OS) task. The final pathomics signature is expressed as a continuous prognostic score calculated using the formula: Pathomics Score = Σ(βi × Xi), where βi represents LASSO‐selected coefficients and Xi represents the aggregated feature values for each prognostic pattern. The non‐zero features identified through LASSO‐Cox regression were incorporated into multivariate Cox models to predict OS and RFS in the training cohort. To stratify patients based on risk, optimal cutoff values for the pathomics signature were determined using X‐tile software (version 3.6.1, Yale School of Medicine), defining high‐ and low‐risk groups. These thresholds were then applied consistently across external validation cohorts to evaluate the model's generalizability. To account for variability across participating centers, a mixed‐effects Cox model was employed, incorporating study site as a random effect. This approach ensured that institutional differences were appropriately accounted for in assessing the prognostic performance of the pathomics signature.

### 5.5 Statistical Analysis

Normality of clinical parameters was assessed using the Shapiro–Wilk test. For group comparisons, independent *t*‐tests were applied to normally distributed variables, while the Mann–Whitney U test was used for non‐normally distributed variables. Categorical variables were compared using Pearson's Chi‐squared test or Fisher's exact test, as appropriate. To evaluate model consistency across centers, we calculated center‐specific performance metrics and conducted formal heterogeneity assessments using meta‐analytic methods with random‐effects modeling. Comparative analyses with established prognostic markers were conducted across all validation cohorts using C‐indices and time‐dependent area under the curve (AUC) analyses. Computational analyses were performed using Python 3.7.12 (incorporating Onekey 3.3.5 and scikit‐learn 1.0.2) and R 4.4.1, with model training carried out on PyTorch 1.8.1 utilizing NVIDIA 4090 graphics processing unit architecture. The discriminative ability of the prognostic classifier was evaluated using Harrell's C‐index. Survival outcomes were analyzed using Kaplan–Meier curves, with statistical differences evaluated by log‐rank tests. Univariate and multivariate Cox proportional hazards models were employed to identify independent prognostic factors. Statistical significance was defined as a two‐sided *p*‐value < 0.05, with Bonferroni correction applied for multiple comparisons.

### 5.6 Ethics Approval and Consent to Participate

The study was approved by the Ethics Committee of Zhongshan Hospital, Fudan University (B2025‐207R), Tianjin Medical University Cancer Institute and Hospital (EK20240007), and the First Affiliated Hospital of Soochow University (EC2025036). All study subjects provided written informed consent. The study was performed in accordance with the Declaration of Helsinki.

## Author Contributions

NP, QC and ZX participated in the conceptualization and design. NP, QC, ZX and ZJ interpretation of the reported experiments or results. All authors participated in the acquisition and analysis of data. NP, JY, LL, JH, QC, ZX and ZJ participated in drafting and revising the manuscript. All the authors revised the manuscript and agreed with the manuscript's results and conclusion. NP supervised the study.

## Conflicts of Interest

The authors declare no conflicts of interest.

## Supporting information




**Supporting File**: advs73658‐sup‐0001‐SuppMat.docx

**Supplementary Table**: advs73658‐sup‐0002‐Supplementary Table S7. Features dataset.xlsx

## Data Availability

The data that support the findings of this study are available from the corresponding author upon reasonable request.

## References

[1] R. L. Siegel , T. B. Kratzer , A. N. Giaquinto , H. Sung , and A. Jemal , “Cancer Statistics,” A Cancer Journal for Clinicians 75 (2025): 10–45, 10.3322/caac.21871. PMC11745215 39817679

[2] L. Rahib , M. R. Wehner , L. M. Matrisian , and K. T. Nead , “Estimated Projection of US Cancer Incidence and Death to 2040,” JAMA Network Open 4 (2021): 214708, 10.1001/jamanetworkopen.2021.4708. PMC8027914 33825840

[3] P. Bailey , D. K. Chang , K. Nones , et al., “Genomic Analyses Identify Molecular Subtypes Of Pancreatic Cancer,” Nature 531 (2016): 47–52, 10.1038/nature16965. 26909576

[4] E. A. Collisson , A. Sadanandam , P. Olson , et al., “Subtypes of Pancreatic Ductal Adenocarcinoma And Their Differing Responses To Therapy,” Nature Medicine 17 (2011): 500–503, 10.1038/nm.2344. PMC3755490 21460848

[5] D. Cui Zhou , R. G. Jayasinghe , S. Chen , et al., “Spatially Restricted Drivers And Transitional Cell Populations Cooperate With the Microenvironment in Untreated And Chemo‐Resistant Pancreatic Cancer,” Nature Genetics 54 (2022): 1390–1405, 10.1038/s41588-022-01157-1. 35995947 PMC9470535

[6] M. M. Chen , Q. Gao , H. Ning , et al., “Integrated Single‐Cell And Spatial Transcriptomics Uncover Distinct Cellular Subtypes Involved In Neural Invasion In Pancreatic Cancer,” Cancer Cell 43 (2025): 1656–1676, 10.1016/j.ccell.2025.06.020. 40680743

[7] C. Liu , S. Deng , K. Jin , et al., “Lewis Antigen‑Negative Pancreatic Cancer: An Aggressive Subgroup,” International Journal of Oncology 56 (2020): 900–908, 10.3892/ijo.2020.4989. 32319567 PMC7050983

[8] R. J. Chen , M. Y. Lu , D. F. K. Williamson , et al., “Pan‐Cancer Integrative Histology‐Genomic Analysis via Multimodal Deep Learning,” Cancer Cell 40 (2022): 865–878, 10.1016/j.ccell.2022.07.004. 35944502 PMC10397370

[9] J. Lee , G. Lee , T.‐Y. Kwak , S. W. Kim , and H. Chang , “Abstract 5060: A Deep Learning Based Pancreatic Adenocarcinoma Survival Prediction Model Applicable To Adenocarcinoma Of Other Organs,” Cancer Research 82 (2022): 5060, 10.1158/1538-7445.AM2022-5060.

[10] P. Vendittelli , J. M. Bokhorst , E. M. M. Smeets , et al., “Automatic Quantification Of Tumor‐Stroma Ratio As A Prognostic Marker For Pancreatic Cancer,” PLoS ONE 19 (2024): 0301969, 10.1371/journal.pone.0301969. PMC11108171 38771787

[11] A. A. Bapat , G. Hostetter , D. D. Von Hoff , and H. Han , “Perineural Invasion and Associated Pain in Pancreatic Cancer,” Nature Reviews Cancer 11 (2011): 695–707, 10.1038/nrc3131. 21941281

[12] J. L. Carstens , P. Correa de Sampaio , D. Yang , et al., “Spatial Computation of Intratumoral T Cells Correlates With Survival Of Patients With Pancreatic Cancer,” Nature Communications 8 (2017): 15095, 10.1038/ncomms15095. PMC5414182 28447602

[13] B. Li , M. S. Nelson , O. Savari , A. G. Loeffler , and K. W. Eliceiri , “Differentiation of pancreatic ductal adenocarcinoma and chronic pancreatitis using graph neural networks on histopathology and collagen fiber features,” Journal of Pathology Informatics 13 (2022): 100158, 10.1016/j.jpi.2022.100158. 36605110 PMC9808020

[14] A. M. Khaliq , M. Rajamohan , O. Saeed , et al., “Spatial Transcriptomic Analysis Of Primary And Metastatic Pancreatic Cancers Highlights Tumor Microenvironmental Heterogeneity,” Nature Genetics 56 (2024): 2455–2465, 10.1038/s41588-024-01914-4. 39294496

[15] K. Cao , Y. Xia , J. Yao , et al., “Large‐Scale Pancreatic Cancer Detection Via Non‐Contrast CT and Deep Learning,” Nature Medicine 29 (2023): 3033–3043, 10.1038/s41591-023-02640-w. PMC10719100 37985692

[16] D. Placido , B. Yuan , J. X. Hjaltelin , et al., “A Deep Learning Algorithm To Predict Risk Of Pancreatic Cancer From Disease Trajectories,” Nature Medicine 29 (2023): 1113–1122, 10.1038/s41591-023-02332-5. PMC10202814 37156936

[17] G. Luo , K. Jin , S. Deng , et al., “Roles of CA19‐9 in Pancreatic Cancer: Biomarker, Predictor and Promoter,” Biochimica Et Biophysica Acta (BBA)—Reviews on Cancer 1875 (1875): 188409, 10.1016/j.bbcan.2020.188409. 32827580

[18] M. Del Chiaro , T. Sugawara , S. D. Karam , and W. A. Messersmith , “Advances in the Management of Pancreatic Cancer,” Bmj 383 (2023): 073995, 10.1136/bmj-2022-073995. 38164628

[19] M. K. K. Niazi , A. V. Parwani , and M. N. Gurcan , “Digital Pathology and Artificial Intelligence,” The Lancet Oncology 20 (2019): e253–e261, 10.1016/s1470-2045(19)30154-8. 31044723 PMC8711251

[20] A. Echle , N. T. Rindtorff , T. J. Brinker , T. Luedde , A. T. Pearson , and J. N. Kather , “Deep Learning in Cancer Pathology: A New Generation Of Clinical Biomarkers,” British Journal of Cancer 124 (2021): 686–696, 10.1038/s41416-020-01122-x. 33204028 PMC7884739

[21] Y. Zhang and J. Yan , 2023 Inter. Conf. on Learning Representations (ICLR) , IEEE 2023, https://openreview.net/forum?id=vSVLM2j9eie.

[22] L. Deininger , B. Stimpel , A. Yüce , et al., A Comparative Study Between Vision Transformers and CNNs in Digital Pathology, arXiv preprint arXiv:2206.00389 2022, 10.48550/ARXIV.2206.00389.

[23] A. Dosovitskiy , L. Beyer , A. Kolesnikov , et al., in 2021 Inter. Conf. on Learning Representations (ICLR), Virtual Event , 2021, https://openreview.net/forum?id=YicbFdNTTy.

[24] E. N. Baruch , F. O. Gleber‐Netto , P. Nagarajan , et al., “Cancer‐induced nerve injury promotes resistance to anti‐PD‐1 therapy,” Nature 646 (2025): 462–473, 10.1038/s41586-025-09370-8. 40836096 PMC12406299

[25] C. Feig , J. O. Jones , M. Kraman , et al., “Targeting CXCL12 From FAP‐Expressing Carcinoma‐Associated Fibroblasts Synergizes With Anti–PD‐L1 Immunotherapy In Pancreatic Cancer,” Proceedings of the National Academy of Sciences 110 (2013): 20212–20217, 10.1073/pnas.1320318110. PMC3864274 24277834

[26] S. M. Liudahl , C. B. Betts , S. Sivagnanam , et al., “Leukocyte Heterogeneity in Pancreatic Ductal Adenocarcinoma: Phenotypic and Spatial Features Associated With Clinical Outcome,” Cancer Discovery 11 (2014): 2014–2031, 10.1158/2159-8290.Cd-20-0841. PMC8338775 33727309

[27] P. P. Provenzano , C. Cuevas , A. E. Chang , V. K. Goel , D. D. Von Hoff , and S. R. Hingorani , “Enzymatic Targeting of the Stroma Ablates Physical Barriers to Treatment of Pancreatic Ductal Adenocarcinoma,” Cancer Cell 21 (2012): 418–429, 10.1016/j.ccr.2012.01.007. 22439937 PMC3371414

[28] K. Y. Aguilera , L. B. Rivera , H. Hur , et al., “Collagen Signaling Enhances Tumor Progression After Anti‐VEGF Therapy in a Murine Model of Pancreatic Ductal Adenocarcinoma,” Cancer Research 74 (2014): 1032–1044, 10.1158/0008-5472.Can-13-2800. 24346431 PMC3944405

[29] M. A. Jacobetz , D. S. Chan , A. Neesse , et al., “Hyaluronan Impairs Vascular Function and Drug Delivery In A Mouse Model of Pancreatic Cancer,” Gut 62 (2013): 112–120, 10.1136/gutjnl-2012-302529. 22466618 PMC3551211

[30] K. H. Chow , R. E. Factor , and K. S. Ullman , “The Nuclear Envelope Environment And Its Cancer Connections,” Nature Reviews Cancer 12 (2012): 196–209, 10.1038/nrc3219. 22337151 PMC4338998

[31] M. Kastinen , P. Sirniö , H. Elomaa , et al., “Immunological and Prognostic Significance of Tumour Necrosis in Colorectal Cancer,” British Journal of Cancer 128 (2023): 2218–2226, 10.1038/s41416-023-02258-2. 37031328 PMC10241859

[32] S. Y. Lee , M. K. Ju , H. M. Jeon , et al., “Regulation of Tumor Progression by Programmed Necrosis,” Oxidative Medicine and Cellular Longevity 2018 (2018): 3537471, 10.1155/2018/3537471. 29636841 PMC5831895

[33] N. Hiraoka , Y. Ino , S. Sekine , et al., “Tumour Necrosis is a Postoperative Prognostic Marker For Pancreatic Cancer Patients With A High Interobserver Reproducibility in Histological Evaluation,” British Journal of Cancer 103 (2010): 1057–1065, 10.1038/sj.bjc.6605854. 20736942 PMC2965866

[34] M. L. Mitchell and C. N. Carney , “Cytologic Criteria for the Diagnosis of Pancreatic Carcinoma,” American Journal of Clinical Pathology 83 (1985): 171–176, 10.1093/ajcp/83.2.171. 2982255

[35] Y. Zhu , B. L. Knolhoff , M. A. Meyer , et al., “CSF1/CSF1R Blockade Reprograms Tumor‐Infiltrating Macrophages and Improves Response to T‐cell Checkpoint Immunotherapy in Pancreatic Cancer Models,” Cancer Research 74 (2014): 5057–5069, 10.1158/0008-5472.Can-13-3723. 25082815 PMC4182950

[36] W. Wang , J. M. Marinis , A. M. Beal , et al., “RIP1 Kinase Drives Macrophage‐Mediated Adaptive Immune Tolerance in Pancreatic Cancer,” Cancer Cell 34 (2018): 757–774, 10.1016/j.ccell.2018.10.006. 30423296 PMC6836726

[37] G. C. Hansson and D. Zopf , “Biosynthesis of the Cancer‐Associated Sialyl‐Lea Antigen,” Journal of Biological Chemistry 260 (1985): 9388–9392, 10.1016/S0021-9258(17)39378-X. 4019478

[38] C. Stossel , M. Raitses‐Gurevich , D. Atias , et al., “Spectrum of Response to Platinum and PARP Inhibitors in Germline BRCA –Associated Pancreatic Cancer in the Clinical and Preclinical Setting,” Cancer Discovery 13 (2023): 1826–1843, 10.1158/2159-8290.Cd-22-0412. 37449843 PMC10401074

[39] R. A. Moffitt , R. Marayati , E. L. Flate , et al., “Virtual Microdissection Identifies Distinct Tumor‐ And Stroma‐Specific Subtypes Of Pancreatic Ductal Adenocarcinoma,” Nature Genetics 47 (2015): 1168–1178, 10.1038/ng.3398. 26343385 PMC4912058

[40] S. Zeng , M. Pöttler , B. Lan , R. Grützmann , C. Pilarsky , and H. Yang , “Chemoresistance in Pancreatic Cancer,” International Journal of Molecular Sciences 20 (2019): 4504, 10.3390/ijms20184504. 31514451 PMC6770382

[41] J. Budczies , D. Kazdal , M. Menzel , et al., “Tumour Mutational Burden: Clinical Utility, Challenges And Emerging Improvements,” Nature Reviews Clinical Oncology 21 (2024): 725–742, 10.1038/s41571-024-00932-9. 39192001

[42] J. M. Ashton , H. Rehrauer , J. Myers , et al., “Comparative Analysis of Single‐Cell RNA Sequencing Platforms and Methods,” Journal of Biomolecular Techniques: JBT 32 (2021): 3fc1f5f3eccea01, 10.7171/3fc1f5fe.3eccea01. PMC9258609 35837267

[43] J. Wang , J. Yang , A. Narang , et al., “Consensus, Debate, and Prospective on Pancreatic Cancer Treatments,” Journal of Hematology & Oncology 17 (2024): 92, 10.1186/s13045-024-01613-x. 39390609 PMC11468220

[44] Q. Chen , H. Yin , Z. Jiang , et al., “Poor Clinical Outcomes And Immunoevasive Contexture in CD161 + CD8 + T Cells Barren Human Pancreatic Cancer,” Journal for ImmunoTherapy of Cancer 12 (2024): 008694, 10.1136/jitc-2023-008694. PMC10966787 38531664

[45] M. Macenko , M. Niethammer , J. S. Marron , et al., in 2009 IEEE Inter Symp on Biomed Imaging (ISBI) , IEEE, 2009, 1107–1110, 10.1109/ISBI.2009.5193250.

[46] K. He , X. Zhang , S. Ren , and J. Sun , in 2016 IEEE Conf. on Computer Vision and Pattern Recognition (CVPR) , IEEE, 2016, 770–778, 10.1109/CVPR.2016.90.

[47] G. Huang , Z. Liu , L. V. D. Maaten , and K. Q. Weinberger , in 2017 IEEE Conf. on Computer Vision and Pattern Recognition (CVPR) , IEEE, 2017, 2261–2269, 10.1109/CVPR.2017.243.

[48] V. Cheplygina , M. de Bruijne , and J. P. W. Pluim , “Not‐So‐Supervised: A Survey of Semi‐Supervised, Multi‐Instance, and Transfer Learning in Medical Image Analysis,” Medical Image Analysis 54 (2019): 280, 10.1016/j.media.2019.03.009. 30959445

