# Supplementary material for: Pathomics Signature for Prognosis and CA19‐9 Interception in Pancreatic Ductal Adenocarcinoma: A Real‐Life, Multi‐Center Study
Source: Adv Sci (Weinh). 2026 Jan 20;13(16):e15952. doi: 10.1002/advs.202515952 (PMC13042562; doi:10.1002/advs.202515952)
Supplement: Supplementary file 1 — Supporting File: advs73658‐sup‐0001‐SuppMat.docx [file ADVS-13-e15952-s001.docx]

**
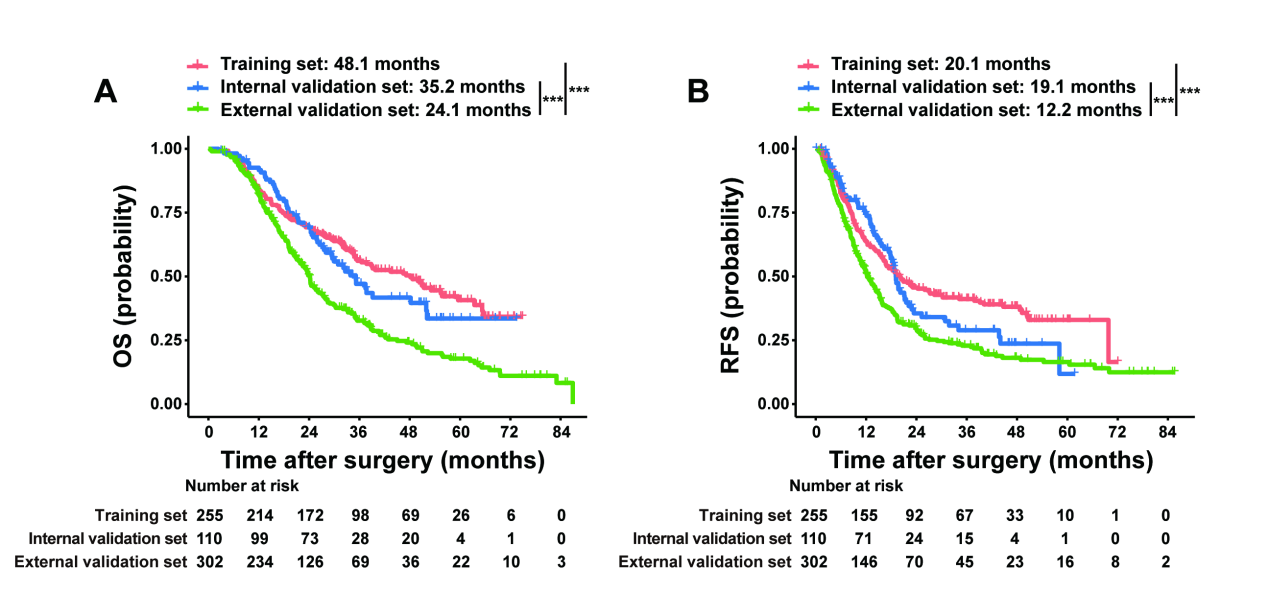
**

**Supplementary Figure S1. Survival analysis for training and validation cohorts.** Kaplan-Meier plots for (A) OS and (B) RFS in the training set, internal validation set and external validation set. OS, overall survival; RFS, recurrence-free survival; ***, p<0.001.


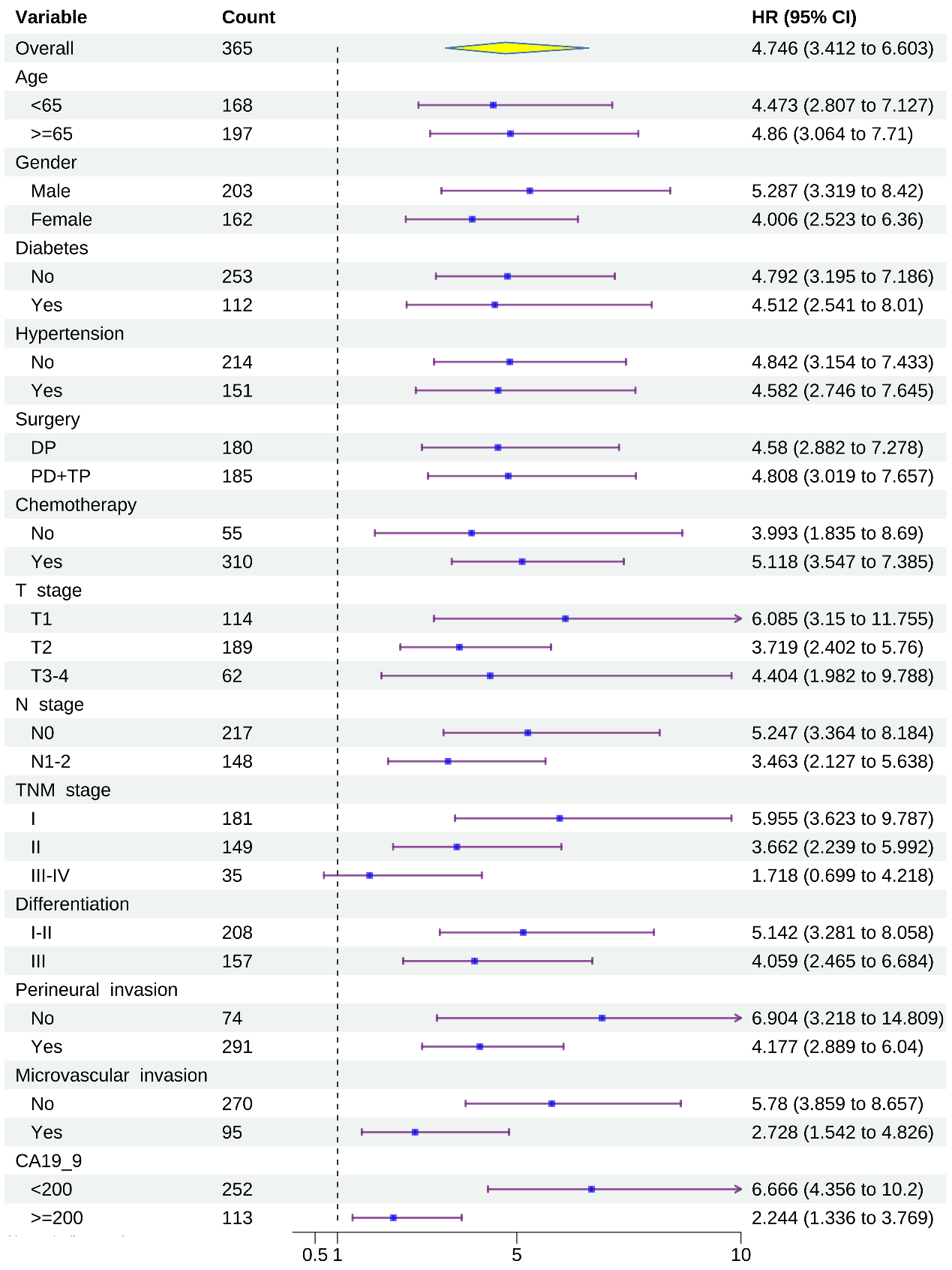


**Supplementary Figure S2. Forest plots of the pathomics signature for OS across prespecified subgroups in the Zhongshan cohort.** Hazard ratios are calculated with the pathomics low-risk group as the reference (HR = 1.0) in all analyses. The forest plots display the relative risk of high-risk versus low-risk patients within each clinical subgroup. The HR and P value were obtained using the Cox proportional hazards model. HR, hazard ratio; CI, confidence interval; OS, overall survival; DP, distal pancreatectomy; PD, pancreaticoduodenectomy; TP, total pancreatectomy.


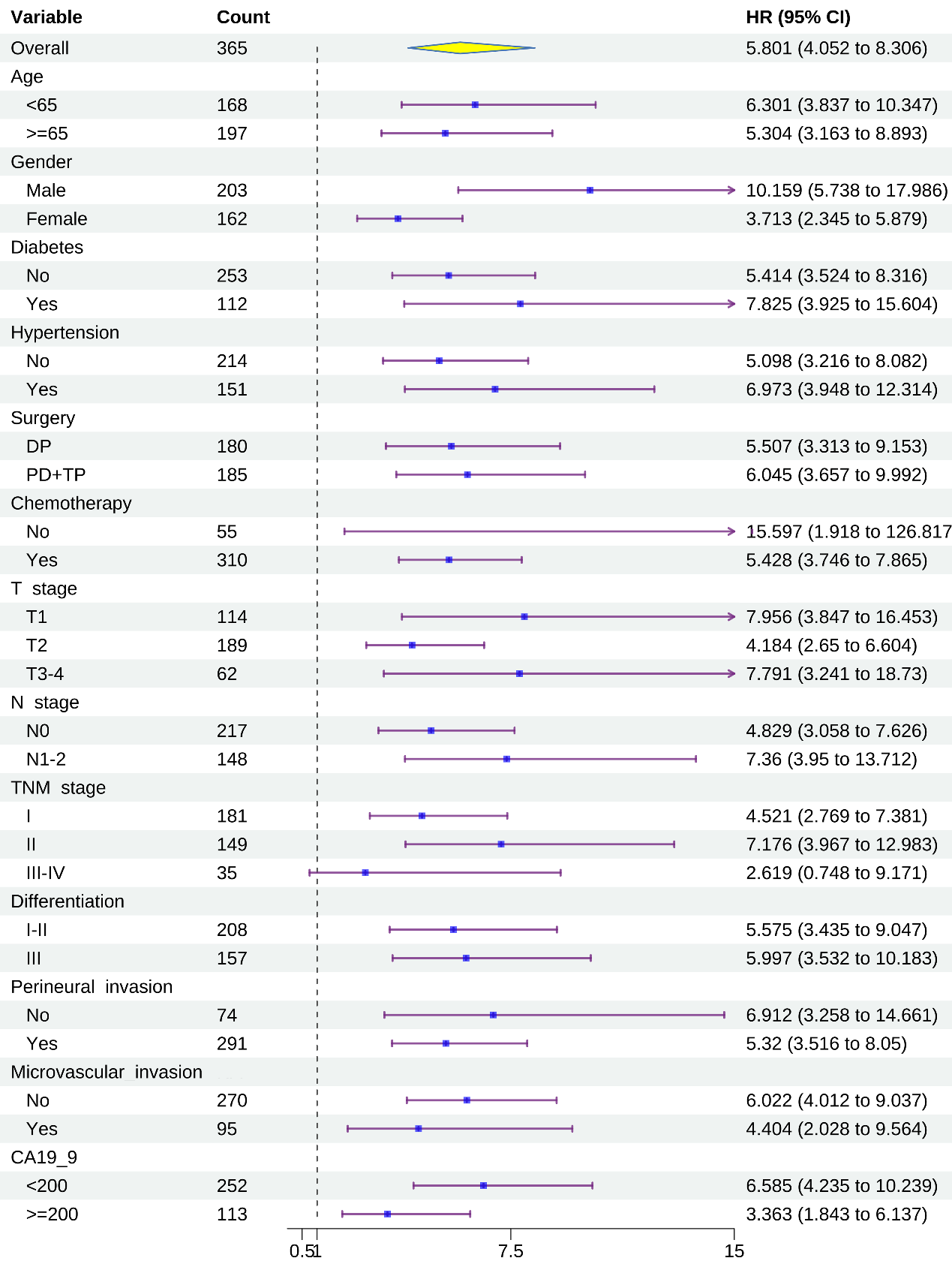


**Supplementary Figure S3. Forest plots of the pathomics signature for RFS across prespecified subgroups in the Zhongshan cohort.** Hazard ratios are calculated with the pathomics low-risk group as the reference (HR = 1.0) in all analyses. The forest plots display the relative risk of high-risk versus low-risk patients within each clinical subgroup. The HR and P value were obtained using the Cox proportional hazards model. HR, hazard ratio; CI, confidence interval; RFS, recurrence-free survival; DP, distal pancreatectomy; PD, pancreaticoduodenectomy; TP, total pancreatectomy.


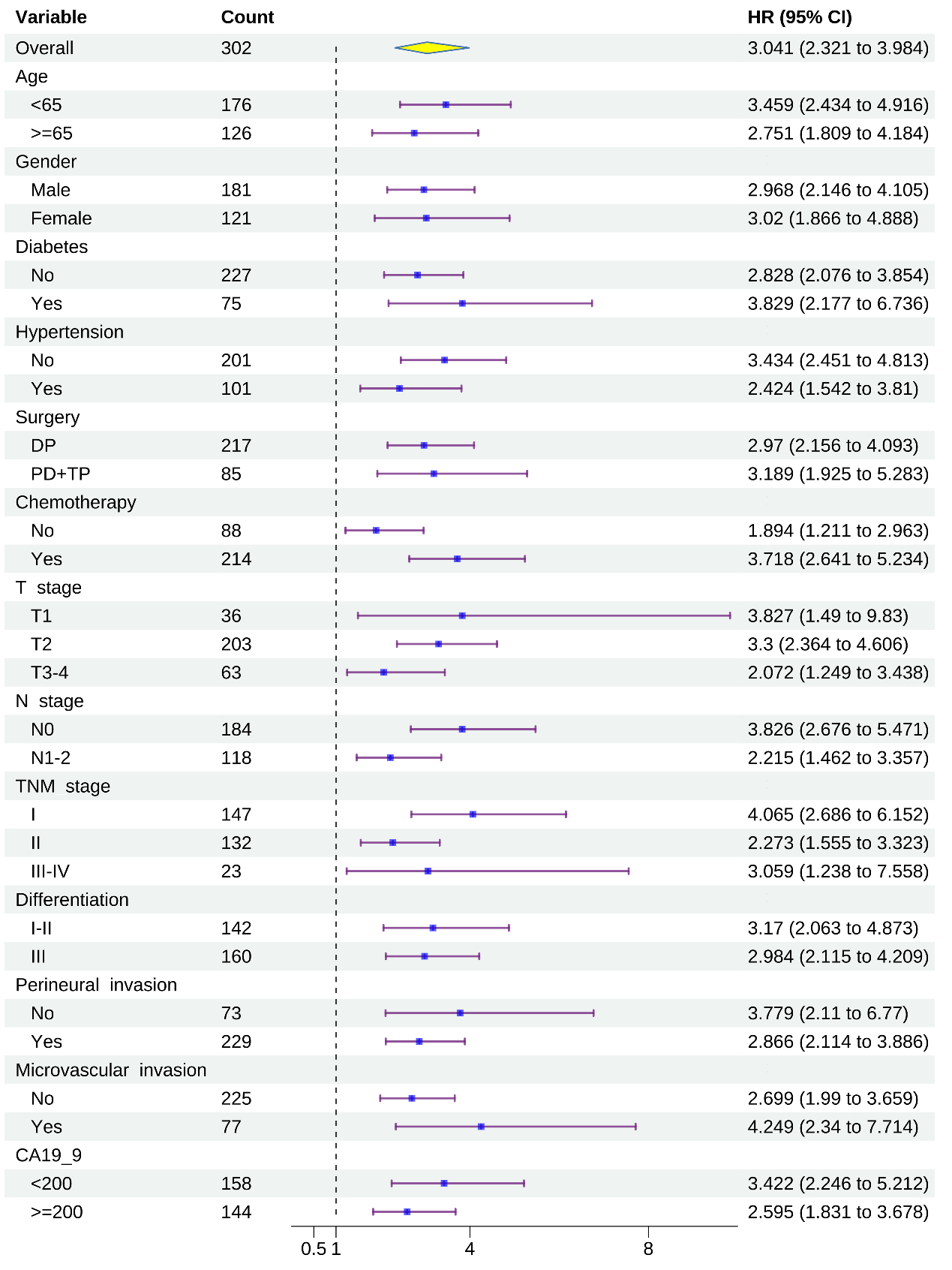


**Supplementary Figure S4. Forest plots of the pathomics signature for OS across prespecified subgroups in the external validation cohort.** Hazard ratios are calculated with the pathomics low-risk group as the reference (HR = 1.0) in all analyses. The forest plots display the relative risk of high-risk versus low-risk patients within each clinical subgroup. The HR and P value were obtained using the Cox proportional hazards model. HR, hazard ratio; CI, confidence interval; OS, overall survival; DP, distal pancreatectomy; PD, pancreaticoduodenectomy; TP, total pancreatectomy.


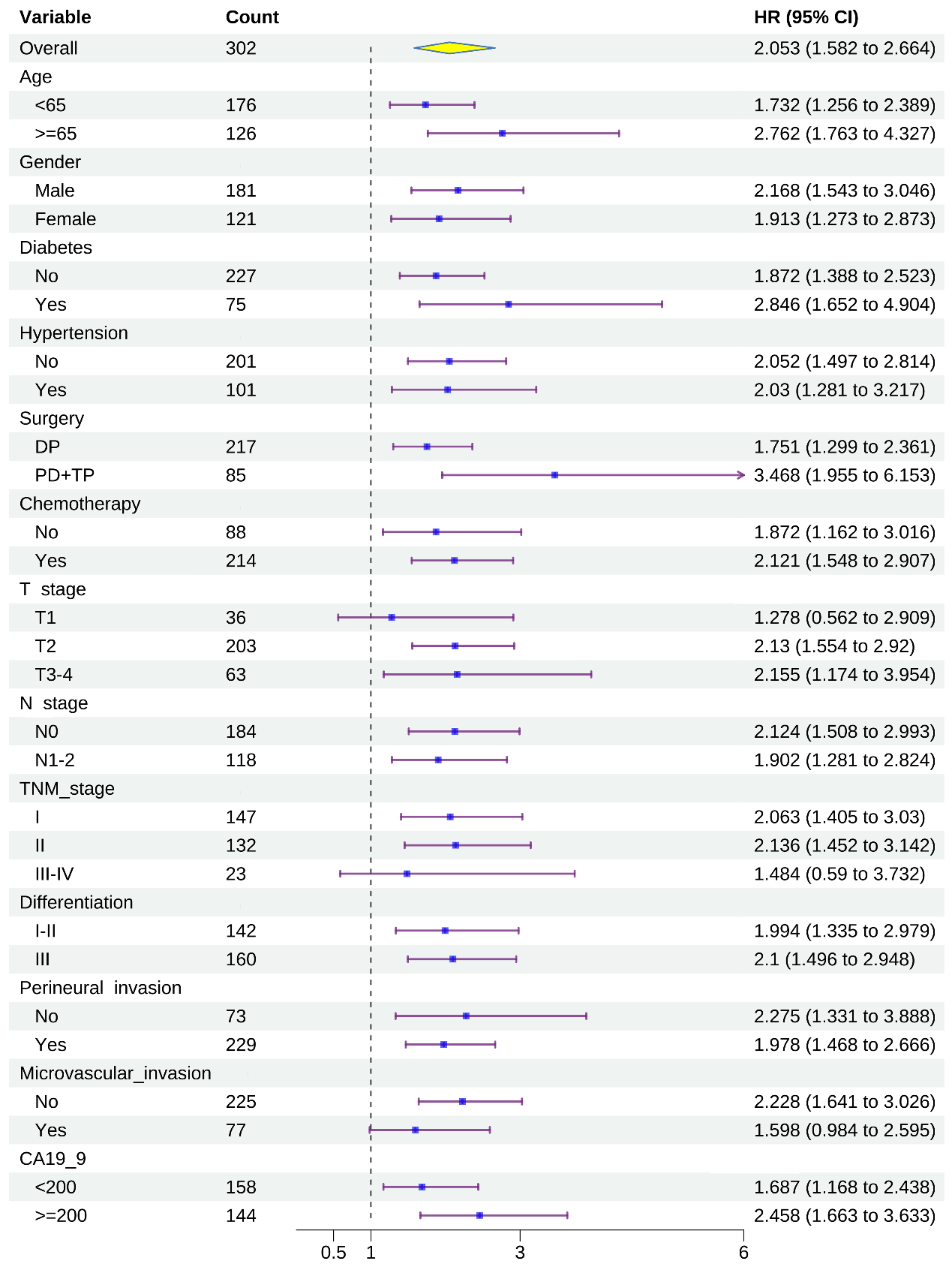


**Supplementary Figure S5. Forest plots of the pathomics signature for RFS across prespecified subgroups in the external validation cohort.** Hazard ratios are calculated with the pathomics low-risk group as the reference (HR = 1.0) in all analyses. The forest plots display the relative risk of high-risk versus low-risk patients within each clinical subgroup. The HR and P value were obtained using the Cox proportional hazards model. HR, hazard ratio; CI, confidence interval; RFS, recurrence-free survival; DP, distal pancreatectomy; PD, pancreaticoduodenectomy; TP, total pancreatectomy.


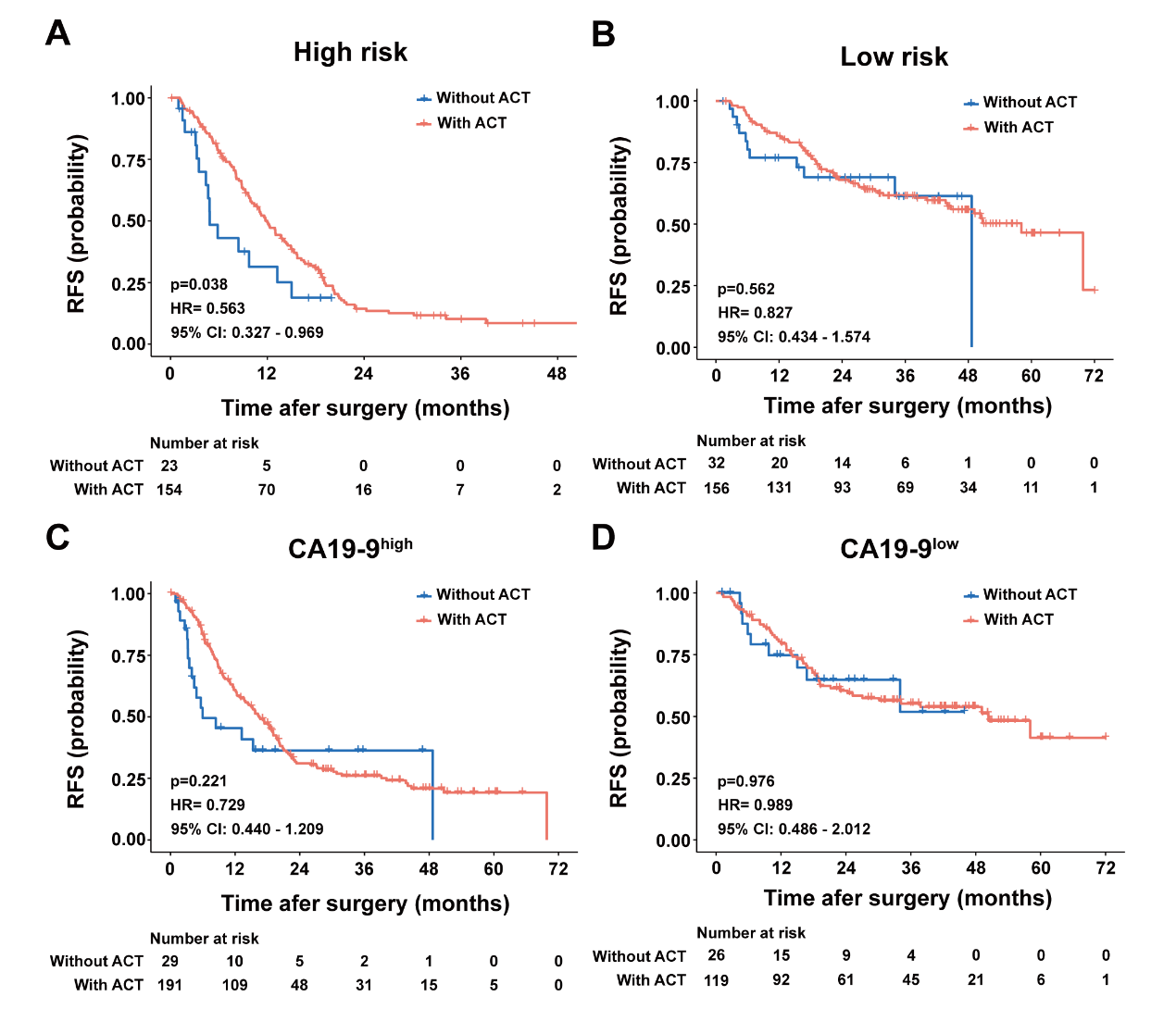


**Supplementary Figure S6. Response to adjuvant chemotherapy stratified by the pathomics signature and CA19-9.** (A, B) Kaplan-Meier analyses of RFS stratified by ACT in the high-risk and low-risk pathomics signature groups. (C, D) Kaplan-Meier analyses of RFS stratified by ACT in the high and low CA19-9 groups. ACT, adjuvant chemotherapy; RFS, recurrence-free survival; HR, hazard ratio; CI, confidence interval.

**Supplementary Table S1. The performance of the different deep learning models across the three independent cohorts.**

| **Model** | **Accuracy** | **Sensitivity** | **Specificity** | **PPV** | **NPV** | **Cohort** |
| --- | --- | --- | --- | --- | --- | --- |
| resnet18  resnet18  resnet18 | 0.713 | 0.672 | 0.753 | 0.727 | 0.700 | Training |
|  | 0.675 | 0.625 | 0.712 | 0.616 | 0.719 | Internal validation |
|  | 0.665 | 0.669 | 0.652 | 0.859 | 0.383 | External validation |
| resnet50 | 0.760 | 0.730 | 0.789 | 0.772 | 0.749 | Training |
| resnet50 | 0.679 | 0.596 | 0.740 | 0.629 | 0.712 | Internal validation |
| resnet50 | 0.657 | 0.639 | 0.713 | 0.876 | 0.384 | External validation |
| densenet121 | 0.753 | 0.716 | 0.790 | 0.770 | 0.739 | Training |
| densenet121 | 0.655 | 0.593 | 0.702 | 0.595 | 0.700 | Internal validation |
| densenet121 | 0.677 | 0.677 | 0.676 | 0.869 | 0.398 | External validation |
| CrossFormer | 0.704 | 0.661 | 0.745 | 0.718 | 0.691 | Training |
| CrossFormer | 0.682 | 0.689 | 0.677 | 0.612 | 0.746 | Internal validation |
| CrossFormer | 0.744 | 0.769 | 0.664 | 0.879 | 0.476 | External validation |

**Supplementary Table S2. Comparative performance of pathomics signatures versus traditional prognostic indicators for recurrence-free survival and overall survival.**

| **Cohort** | **Predictor** | **Recurrence-free survival** | | | | **Overall survival** | | | |
| --- | --- | --- | --- | --- | --- | --- | --- | --- | --- |
|  |  | 1-yr AUC | 2-yr AUC | 3-yr AUC | C-index (95% CI) | 1-yr AUC | 3-yr AUC | 5-yr AUC | C-index (95% CI) |
| **Training Set**  (n=255) | Pathomics | 0.807 | 0.874 | 0.883 | 0.752 (0.712-0.792) | 0.724 | 0.844 | 0.900 | 0.742 (0.736-0.748) |
|  | TNM Staging | 0.628 | 0.633 | 0.638 | 0.594 (0.551-0.636) | 0.664 | 0.575 | 0.598 | 0.585 (0.579-0.592) |
|  | CA19-9 | 0.619 | 0.674 | 0.648 | 0.562 (0.523-0.600) | 0.611 | 0.657 | 0.562 | 0.555 (0.548-0.561) |
|  |  |  |  |  |  |  |  |  |  |
| **Internal Validation Set**  (n=110) | Pathomics | 0.686 | 0.896 | 0.934 | 0.703 (0.640-0.766) | 0.730 | 0.851 | 0.866 | 0.743 (0.730-0.757) |
|  | TNM Staging | 0.606 | 0.742 | 0.750 | 0.620 (0.557-0.684) | 0.834 | 0.654 | 0.476 | 0.659 (0.644-0.673) |
|  | CA19-9 | 0.668 | 0.617 | 0.620 | 0.596 (0.537-0.656) | 0.643 | 0.508 | 0.672 | 0.581 (0.566-0.596) |
|  |  |  |  |  |  |  |  |  |  |
| **External Validation Set**  (n=302) | Pathomics | 0.769 | 0.709 | 0.691 | 0.673 (0.642-0.704) | 0.702 | 0.733 | 0.731 | 0.651 (0.646-0.656) |
|  | TNM Staging | 0.584 | 0.559 | 0.571 | 0.553 (0.516-0.590) | 0.619 | 0.529 | 0.581 | 0.552 (0.547-0.557) |
|  | CA19-9 | 0.613 | 0.595 | 0.637 | 0.568 (0.534-0.602) | 0.587 | 0.592 | 0.563 | 0.551 (0.546-0.556) |

AUC: Area Under the Curve; CI: Confidence Interval; C-index: Concordance Index; CA19-9: Carbohydrate Antigen 19-9; TNM: Tumor, Node, Metastasis staging system. All pathomics signatures demonstrated superior discriminative performance compared to traditional prognostic indicators across all validation cohorts.

**Supplementary Table S3. Univariate and multivariate analysis of prognostic indicators associated with overall survival and recurrence-free survival in the training cohort.**

| **Variables** | **Overall survival** | | | **Recurrence-free survival** | | |
| --- | --- | --- | --- | --- | --- | --- |
|  | **Univariate *P* value** | **Multivariate *P* value** | **Multivariate HR (95% CI)** | **Univariate *P* value** | **Multivariate *P* value** | **Multivariate HR (95% CI)** |
| **Gender** |  |  |  |  |  |  |
| Male/ Female | 0.765 | NA |  | 0.399 | NA |  |
| **Age (years)** |  |  |  |  |  |  |
| <65/ ≥ 65 | 0.907 | NA |  | 0.362 | NA |  |
| **Surgery** |  |  |  |  |  |  |
| PD/ DP /TP | 0.602 | NA |  | 0.439 | NA |  |
| **Differentiation** |  |  |  |  |  |  |
| I-II/ III | **0.003** | 0.085 | 1.393 (0.955 to 2.030) | **0.008** | 0.095 | 1.338 (0.950 to 1.883) |
| **T stage** |  |  |  |  |  |  |
| T1/T2/ T3-4 | **<0.001** | **0.008** | 1.418 (1.096 to 1.834) | **<0.001** | **0.021** | 1.322 (1.043 to 1.677) |
| **N stage** |  |  |  |  |  |  |
| N0/N1/N2  **M stage**  M0/ M1 | **<0.001**  **<0.001** | **0.050**  **0.029** | 1.342 (0.999 to 1.801)  2.388 (1.093 to 5.216) | **<0.001**  **<0.001** | **0.011**  **0.011** | 1.415 (1.084 to 1.847)  2.439 (1.222 to 4.870) |
| **Chemotherapy** |  |  |  |  |  |  |
| No / Yes | **0.003** | **<0.001** | 0.319 (0.194 to 0.524) | 0.289 | NA |  |
| **Diabetes** |  |  |  |  |  |  |
| No / Yes  **Hypertension**  No / Yes | **0.038**  0.887 | 0.489  NA | 1.147 (0.778 to 1.692) | 0.114  0.774 | NA  NA |  |
| **Microvascular invasion** |  |  |  |  |  |  |
| No / Yes | **0.001** | 0.094 | 1.393 (0.945 to 2.054) | **0.013** | 0.715 | 1.071 (0.741-1.547) |
| **Perineural invasion** |  |  |  |  |  |  |
| No / Yes | 0.058 | NA |  | 0.051 | NA |  |
| **CA19-9** |  |  |  |  |  |  |
| <200 / ≥ 200 | **0.005** | 0.118 | 1.339 (0.929 to 1.930) | **<0.001** | 0.119 | 1.307 (0.934 to 1.829) |
| **Pathomics-OS** |  |  |  |  |  |  |
| Low-risk / High-risk | **<0.001** | **<0.001** | 4.494 (2.988 to 6.760) |  |  |  |
| **Pathomics-RFS** |  |  |  |  |  |  |
| Low-risk / High-risk |  |  |  | **<0.001** | **<0.001** | 5.293 (3.365 to 8.325) |

Abbreviations: DP, distal pancreatectomy; PD, pancreaticoduodenectomy; TP, total pancreatectomy.

**Supplementary Table S4. Univariate and multivariate analysis of prognostic indicators associated with overall survival and recurrence-free survival in the internal validation cohort.**

| **Variables** | **Overall survival** | | | **Recurrence-free survival** | | |
| --- | --- | --- | --- | --- | --- | --- |
|  | **Univariate *P* value** | **Multivariate *P* value** | **Multivariate HR (95% CI)** | **Univariate *P* value** | **Multivariate *P* value** | **Multivariate HR (95% CI)** |
| **Gender** |  |  |  |  |  |  |
| Male/ Female | 0.995 | NA |  | 0.632 | NA |  |
| **Age (years)** |  |  |  |  |  |  |
| <65/ ≥ 65 | 0.718 | NA |  | 0.733 | NA |  |
| **Surgery** |  |  |  |  |  |  |
| PD/ DP /TP | 0.673 | NA |  | 0.793 | NA |  |
| **Differentiation** |  |  |  |  |  |  |
| I-II/ III | 0.165 | NA |  | 0.097 | NA |  |
| **T stage** |  |  |  |  |  |  |
| T1/T2/ T3-4 | **0.002** | 0.639 | 1.108 (0.722 to 1.701) | **0.044** | 0.458 | 1.154 (0.791 to 1.683) |
| **N stage** |  |  |  |  |  |  |
| N0/N1/N2  **M stage**  M0/ M1 | **<0.001**  **0.034** | **0.005**  0.107 | 1.972 (1.223 to 3.178)  3.414 (0.767 to 15.195) | **<0.001**  0.886 | **0.002**  NA | 1.966 (1.290 to 2.995) |
| **Chemotherapy** |  |  |  |  |  |  |
| No / Yes | 0.771 | NA |  | 0.438 | NA |  |
| **Diabetes** |  |  |  |  |  |  |
| No / Yes  **Hypertension**  No / Yes | 0.283  0.795 | NA  NA |  | 0.122  0.849 | NA  NA |  |
| **Microvascular invasion** |  |  |  |  |  |  |
| No / Yes | 0.405 | NA |  | 0.100 | NA |  |
| **Perineural invasion** |  |  |  |  |  |  |
| No / Yes | 0.125 | NA |  | 0.095 | NA |  |
| **CA19-9** |  |  |  |  |  |  |
| <200 / ≥ 200 | **0.028** | 0.942 | 1.022 (0.575 to 1.816) | **0.002** | **0.018** | 1.942 (1.123 to 3.359) |
| **Pathomics-OS** |  |  |  |  |  |  |
| Low-risk / High-risk | **<0.001** | **<0.001** | 4.011 (2.084 to 7.720) |  |  |  |
| **Pathomics-RFS** |  |  |  |  |  |  |
| Low-risk / High-risk |  |  |  | **<0.001** | **<0.001** | 4.053 (2.058 to 7.980) |

Abbreviations: DP, distal pancreatectomy; PD, pancreaticoduodenectomy; TP, total pancreatectomy.

**Supplementary Table S5. Univariate and multivariate analysis of prognostic indicators associated with overall survival and recurrence-free survival in the external validation cohort.**

| **Variables** | **Overall survival** | | | **Recurrence-free survival** | | |
| --- | --- | --- | --- | --- | --- | --- |
|  | **Univariate *P* value** | **Multivariate *P* value** | **Multivariate HR (95% CI)** | **Univariate *P* value** | **Multivariate *P* value** | **Multivariate HR (95% CI)** |
| **Gender** |  |  |  |  |  |  |
| Male/ Female | 0.735 | NA |  | 0.681 | NA |  |
| **Age (years)** |  |  |  |  |  |  |
| <65/ ≥ 65 | 0.122 | NA |  | **0.022** | **0.031** | 0.739 (0.561 to 0.972) |
| **Surgery** |  |  |  |  |  |  |
| PD/ DP /TP | 0.747 | NA |  | 0.495 | NA |  |
| **Differentiation** |  |  |  |  |  |  |
| I-II/ III | **0.006** | **0.006** | 1.472 (1.117 to 1.940) | **0.001** | **0.006** | 1.454 (1.115 to 1.895) |
| **T stage** |  |  |  |  |  |  |
| T1/T2/ T3-4 | 0.156 | NA |  | 0.259 | NA |  |
| **N stage** |  |  |  |  |  |  |
| N0/N1/N2  **M stage**  M0/ M1 | 0.135  **0.003** | NA  **0.042** | 3.393 (1.045 to 11.010) | **0.016**  **<0.001** | 0.084  **0.002** | 1.200 (0.976 to 1.476)  6.598 (2.031 to 21.437) |
| **Chemotherapy** |  |  |  |  |  |  |
| No / Yes | **0.002** | **0.034** | 0.729 (0.545 to 0.976) | 0.142 | NA |  |
| **Diabetes** |  |  |  |  |  |  |
| No / Yes  **Hypertension**  No / Yes | 0.805  0.774 | NA  NA |  | 0.485  0.947 | NA  NA |  |
| **Microvascular invasion** |  |  |  |  |  |  |
| No / Yes | 0.669 | NA |  | 0.314 | NA |  |
| **Perineural invasion** |  |  |  |  |  |  |
| No / Yes | 0.240 | NA |  | 0.113 | NA |  |
| **CA19-9** |  |  |  |  |  |  |
| <200 / ≥ 200 | **0.026** | 0.170 | 1.211 (0.921 to 1.593) | **0.002** | **0.015** | 1.397 (1.067 to 1.829) |
| **Pathomics-OS** |  |  |  |  |  |  |
| Low-risk / High-risk | **<0.001** | **<0.001** | 2.763 (1.974 to 3.868) |  |  |  |
| **Pathomics-RFS** |  |  |  |  |  |  |
| Low-risk / High-risk |  |  |  | **<0.001** | **<0.001** | 1.939 (1.476 to 2.546) |

Abbreviations: DP, distal pancreatectomy; PD, pancreaticoduodenectomy; TP, total pancreatectomy.

**Supplementary Table S6. Relationships between the pathomics signature and clinicopathological characteristics in the Zhongshan cohort.**

| **Variables** | **Low-risk group** | **High-risk group** | ***P* value** |
| --- | --- | --- | --- |
|  | **(N=188)** | **(N=177)** |  |
| **Gender** |  |  | 0.026 |
| Female  Male | 94 (50%)  94 (50%) | 109 (62%)  68 (38%) |  |
| **Age**  <65  >=65 | 88 (47%)  100 (53%) | 80 (45%)  97 (55%) | 0.758 |
| **Surgery** |  |  | 0.787 |
| DP  PD + TP | 94 (50%)  94 (50%) | 86 (49%)  91(51%) |  |
| **Differentiation** |  |  | 0.006 |
| I-II  III | 120 (64%)  68 (36%) | 88 (50%)  89 (50%) |  |
| **T stage** |  |  | <0.001 |
| T1  T2  T3-4 | 76 (40%)  90 (48%)  22 (12%) | 38 (21%)  99 (56%)  40 (23%) |  |
| **N stage** |  |  | <0.001 |
| N0  N1-2  **M stage**  M0  M1 | 129 (69%)  59 (31%)  185 (98.4%)  3 (1.6%) | 88 (50%)  89 (50%)  169 (95.5%)  8 (4.5%) | 0.102 |
| **TNM stage**  I  II  III + IV | 113 (60%)  64 (34%)  11 (6.0%) | 68(38%)  85 (48%)  24 (14%) | <0.001 |
| **Adjuvant chemotherapy** |  |  | 0.282 |
| No  Yes | 32 (17%)  156 (83%) | 23 (13%)  154 (87%) |  |
| **Diabetes** |  |  | 0.402 |
| No  Yes | 134 (71%)  54 (29%) | 119 (67%)  58 (33%) |  |
| **Hypertension** |  |  | 0.493 |
| No  Yes | 107 (57%)  81 (43%) | 107 (60%)  70 (40%) |  |
| **Microvascular invasion** |  |  | 0.058 |
| No  Yes | 147 (78%)  41 (22%) | 123 (69%)  54 (31%) |  |
| **Perineural invasion** |  |  | 0.040 |
| No  Yes | 46 (24%)  142 (76%) | 28 (16%)  149 (84%) |  |
| **CA19-9**  **<200**  **>=200** | 142 (76%)  46 (24%) | 110 (62%)  67 (38%) | 0.006 |

Abbreviations: DP, distal pancreatectomy; PD, pancreaticoduodenectomy; TP, total pancreatectomy.
